# Myrislignan Induces Redox Imbalance and Activates Autophagy in *Toxoplasma gondii*

**DOI:** 10.3389/fcimb.2021.730222

**Published:** 2021-09-03

**Authors:** Jili Zhang, Jia Chen, Kun Lv, Bing Li, Biqing Yan, Lei Gai, Chaolu Shi, Xinnian Wang, Hongfei Si, Jiyu Zhang

**Affiliations:** ^1^Intensive Care Unit, The Affiliated Hospital of Medical School, Ningbo University, Ningbo, China; ^2^Ningbo University School of Medicine, Ningbo University, Ningbo, China; ^3^Ningbo University School of Business, Ningbo University, Ningbo, China; ^4^Lanzhou Institute of Husbandry and Pharmaceutical Sciences, Chinese Academy of Agricultural Sciences, Lanzhou, China; ^5^College of Pharmacy, Jinan University, Guangzhou, China

**Keywords:** myrislignan, *Toxoplasma gondii*, oxidation-reduction process, autophagy, oxidative phosphorylation

## Abstract

*Toxoplasma gondii (T. gondii)* is an important health problem in human and animals, and the highlighting side effects of launched therapeutic chemicals cannot be ignored. Thus, it is urgent to develop new drugs to against the infection. Myrislignan originated from nutmeg exhibited excellent anti-*T. gondii* activity *in vitro* and *in vivo*, and was able to destroy mitochondrial function. However, the exact mechanism of action is still unknown. In this study, combining RNAs deep-sequencing analysis and surface plasmon resonance (SPR) analysis, the differentially expressed genes (DEGs) and high affinity proteins suggested that myrislignan may affect the oxidation-reduction process of *T. gondii*. Furthermore, the upregulating ROS activity after myrislignan incubation verified that myrislignan destroyed the oxidant-antioxidant homeostasis of tachyzoites. Transmission electron microscopy (TEM) indicated that myrislignan induced the formation of autophagosome-like double-membrane structure. Moreover, monodansyl cadaverine (MDC) staining and western blot further illustrated autophagosome formation. Myrislignan treatment induced a significant reduction in *T. gondii by* flow cytometry analysis. Together, these findings demonstrated that myrislignan can induce the oxidation-reduction in *T. gondii*, lead to the autophagy, and cause the death of *T. gondii.*

## Introduction

*Toxoplasma gondii* (*T. gondii*) is a kind of parasites that causes widespread zoonotic toxoplasmosis by affecting human health and disrupting animal husbandry. It is a critical public health burden that has caused global concerns (Weiss and Kim, 2013). The general affective symptoms are not obvious in immunocompetent individuals. However, as for immune-compromised individuals, especially in AIDS, *T. gondii* infection often caused serious consequences (Ahmadpour et al., 2014). Furthermore, during pregnancy, *T. gondii* infection through vertical transmission can result in miscarriage, foetal malformations or even death (Fallahi et al., 2018). Currently, pyrimethamine and sulfadiazine are the gold standard therapeutic drugs (Giovati et al., 2018). However, these therapeutic treatments remain dissatisfactory effects because of significant bone marrow toxicity, drug toxicity and failure to against latent infections. Therefore, novel therapeutic drugs are in urgent need for future intervention strategies.

Constant efforts have been made to seek anti-parasitic drugs against zoonotic parasitic disease, but the novel anti-*T. gondii* drugs with high effectiveness and low toxicity have not yet been launched (Choi and Lee, 2019). It is worth noting that natural products from plants are useful source for developing the anti-*T. gondii* drugs. Myrislignan is a natural product from *Myristica fragrans* Houtt with a wide range of pharmacological activities (Nguyen et al., 2010; Jin et al., 2012; Lu et al., 2017; Yang et al., 2018; Zhang et al., 2019). In the previous study, we have demonstrated that myrislignan could inhibit *T. gondii* replication and invasion in *T. gondii in vitro* without affecting the host cells. Furthermore, myrislignan exposure also induced the surface shrinkage and mitochondrial damage in *T. gondii*. Despite the mitochondrial damage has been further confirmed by the reduced ΔΨm and ATP levels in tachyzoites treated with myrislignan, it is also well worth investigating the mechanism of action of myrislignan against *T. gondii*, thereby highlighting its therapeutic potential in toxoplasmosis. Herein, we illustrated myrislignan may affect the oxidant-antioxidant homeostasis of *T. gondii* and cause autophagy of *T. gondii*, and lead to programmed death of *T. gondii*.

## Materials and Methods

### Cells and Parasites

African green monkey kidney (Vero) cells were cultured in Dulbecco’s Modified Eagle’s Medium (DMEM) supplemented with 10% (v/v) heat-inactivated foetal bovine serum (FBS), 1% non-essential amino acids (NEAA), 100 U/mL penicillin, 100 μg/mL streptomycin and 1% GlutaMAX at 37°C in a 5% CO_2_ atmosphere (Zhang et al., 2019). The *T. gondii* RH stain tachyzoites used in our study were maintained in Vero layers in DMEM contained with 1% FBS, as described previously (Si et al., 2018). All the infection experiments with *T. gondii* were performed under biosafety level 2 (BSL-2) conditions.

### Drugs

Myrislignan (batch numbers DST180502-043, Desite Biotechnology Co., Ltd., China) was dissolved in dimethyl sulfoxide (DMSO, Sigma, USA) at a concentration of 4 mg/mL, then diluted in DMEM containing 1% FBS to different concentrations. All drugs were stored at 4°C.

### RNA Preparation and Sequencing

*T. gondii* were isolated from infected Vero cells according to previously described methods. After treatment with different concentrations of myrislignan (32, 50 or 70 μg/mL) in DMEM or without any drug (as parasite control) for 24 h at 37°C, all the samples were washed with cold phosphate-buffered saline (PBS) and immediately stored at -80°C until they were used for RNA isolation. RNA-Seq analysis was based on three biological replicates per experimental group. Total RNA was extracted from *T. gondii* using TRIzol Reagent (Invitrogen, USA) and the concentrations were detected by an Agilent 2100 Bioanalyzer (Agilent RNA 6000 Nano Kit, Agilent Technologies, USA). Sequencing libraries were generated using an Illumina TruSeqTM RNA Sample Preparation Kit (Illumina, San Diego, CA, USA) and sequenced with the HiSeq 2000 System (TruSeq SBS KIT-HS V3, Illumina). RNA isolation, library construction, RNA sequencing, and read alignment were performed by BGI (Shenzhen, China) (He et al., 2019).

The level of gene expression was calculated in units of fragments per kilobase of transcript sequence per million base pairs sequenced (FPKM) of each gene. Differential expression analysis was performed using the DESeq R package. The P-values were adjusted as Q-values using the Benjamini-Hochberg and Storey-Tibshirani correction for multiple testing. As the |log2 (fold change)| ≥ 1 and Q-values ≤ 0.001, the transcripts were considered differentially expressed. DEGs were subjected to Gene Ontology (GO) (www.geneontology.org/) enrichment and Kyoto Encyclopedia of Genes and Genomes (KEGG) (www.genome.jp/kegg/) analyses, which were performed as described previously (He et al., 2019).

### Validation of mRNA Expression

Total RNA from treated and untreated *T. gondii* tachyzoites was extracted as described above, then used to synthesized cDNA. TB Green Premix Ex Taq II (Tli RNaseH Plus) (TaKaRa, Japan) was used to perform quantitative real-time polymerase chain reaction (qRT-PCR) reactions using a QuantStudio 6 Flex Real-Time PCR System (Life Technologies). The qRT-PCR primers used in this study are described in **Supplementary Dataset S1**. α-tubulin was used as an internal standard reference gene. Each sample were carried out in biological triplicates.

### Surface Plasmon Resonance (SPR) Experiment

3.74 mg/mL myrislignan in DMSO were spotted in 3D SPRi chips using a BioDot 1520 Array Printer to control the consistency of sample size. No myrislignan in DMSO were spotted in chips as the negative control spot. Freshly released *T. gondii* tachyzoites (1×10^9^) were lysed, and the protein concentration was detected with a Thermo Fisher BCA Protein Assay Kit (Number: 23227). The final concentration of the *T. gondii* sample was 200 µg/mL. Protein lysate was flowed through the chip surface to bind the compound on the chip surface, and PBST was also used as the negative control for the measurement of specific signals in oval regions of interest. After *in situ* enzymatic hydrolysis, the kinetic affinity between *T. gondii* peptides and myrislignan was calculated, and the protein or peptides captured on the chip surface were identified by HPLC-MS/MS (Nano Acquity UPLC System, Waters Corp., USA; AB SCIEX TOF/TOF Mass Spectrometry System, AB Sciex Pte. Ltd, USA).

### The Reactive Oxygen Species (ROS) Production

Tachyzoites in Vero cells were treated with myrislignan (32 or 70 μg/mL) in DMEM or with no drug (as control) for 8 h, 16 h or 24 h, then fresh tachyzoites (approximately 1×10^6^/group) were extracted and incubated with 10 µM H2DCFDA (DCFH-DA, 2’,7’-Dichlorodihydrofluorescein diacetate) probe in DMEM for 20 min at 37°C. All the samples were washed with DMEM twice, and seeded into each well of a 24-well cell culture plate, then the luminescence was detected using a multilabel reader (EnSpire, PerkinElmer, USA) (Chen et al., 2018).

### Superoxide Dismutase (SOD) Activity

Fresh tachyzoites (1×10^6^ per group) from Vero cells were lysed after incubation with myrislignan (32 or 70 μg/mL) or without drug (control), then centrifuged at 12,000 g for 5 min at 4°C. The supernatant was added to a 96 well plates, and the absorbance of each sample was measured at 450 nm by a total SOD assay kit (WST-8, Beyotime, China) after incubation at 37°C for 30 min in dark (Chen et al., 2018).

### Transmission Electron Microscopy (TEM) Analysis

Vero infected with *T. gondii* for 8 h and incubated with 32 or 70 μg/mL myrislignan for 16 h or 24 h, digested with TrypLE Express for 2 min, washed twice with PBS. Then, the cells were processed for TEM, as described previously (Si et al., 2018).

### Monodansyl Cadaverine (MDC) Detection

For each sample, tachyzoites in Vero cells were treated with myrislignan (32 or 70 μg/mL) for 16 h in DMEM or with no drug (as a control). After extraction, the fresh tachyzoites were suspended in the MDC solution (100 μM) at 37°C for 60 min, and then washed with PBS, resuspended in 500 μL PBS. The fluorescence in each group was visualized by laser scanning confocal microscopy (ZEISS LSM-800, Jena, German). The experiment was repeated three times (Zhang et al., 2021).

### Western Blotting Analysis

After myrislignan (16, 32, 50, 60 or 70 μg/mL) treatment for 16 h, *T. gondii* were lysed with RIPA lysis buffer, all protein samples were separated on 15% urea SDS-polyacrylamide gel electrophoresis and transferred onto 0.22 μm polyvinylidene fluoride (PVDF) membranes (Merck Millipore, US) (Besteiro et al., 2011; Kong-Hap et al., 2013). After blocking, membranes were incubated with the corresponding primary antibodies against TgATG8 (1: 250, presented by researcher Dr. Jia of Harbin Institute of Veterinary Medicine) at 4°C overnight. The membranes were washed with TBST buffer and incubated with horseradish peroxidase-conjugated secondary antibodies (1:2000, Cell Signaling Technology, USA), and chemiluminescent detection was completed with enhanced chemiluminescence Western blot agent (Millipore, Billerica, MA, USA). The protein signals were detected with Amersham Imager 600 system (GE, Boston, MA, USA) and were normalized to the corresponding internal control tubulin to eliminate the variance in total protein (Wang et al., 2010; Lee et al., 2013).

### Flow Cytometry Analysis

*T. gondii* tachyzoites in infected host cells and incubation with myrislignan (32, 50 or 70 μg/mL) for 24 h. Intracellular parasites were collected by passage of host cells, and approximately 1×10^6^ tachyzoites were centrifugation at 1,500 g, 15 min at 4°C and washed with PBS. Then, the samples were suspended in 100 μL of binding buffer with 5 μL of annexin V-PE and 5 μL of 7-AAD dye (Becton Dickinson Company, 559763) in the dark for 20 min at 37°C. Double mixtures were analysed by Guava easyCyte flow cytometer (Merck, USA) (Chen et al., 2018). The experiment was repeated three times.

### Statistical Analyses

Data comparisons between the control and myrislignan treatment groups in the ROS and SOD tests, flow cytometry assay was statistically analysed by one-way analysis of variance (ANOVA) using SPSS software (SPSS Inc., Chicago, IL, USA). Differences were considered statistically significant at a *p* value <0.01.

## Results

### RNA-Seq Data Analysis and Verification

**Figure 1:**
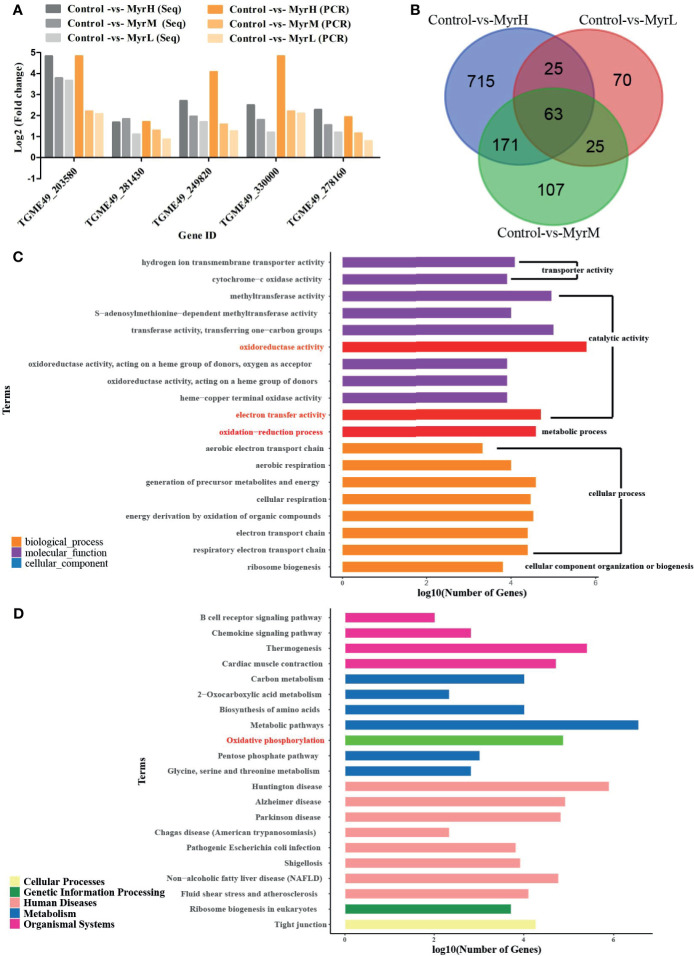
RNA-Seq data analysis and verification. Verification the RNA-Seq data by qRT-PCR **(A)** MyrH (Myrislignan 70 μg/mL); MyrM (Myrislignan 50 μg/mL); MyrL (Myrislignan 32 μg/mL); Venn diagram analysis revealed the DEGs in myrislignan-treated groups (32, 50 or 70 μg/mL) compared with those in the control-treated groups **(B)** GO enrichment analysis of DEGs in *T. gondii* after treatment with myrislignan, DEGs were sorted into three categories: cellular component, biological process and molecular function **(C)** KEGG pathway analysis of RNA-Seq data, among which the x-axis shows the Log10 (number of gene) and the y-axis corresponds to KEGG pathway **(D)**. MyrH (Myrislignan high dose group); MyrM (Myrislignan middle dose group); MyrL (Myrislignan low dose group).

RNA-Seq was used to investigate the gene expression patterns of *T. gondii* treated with myrislignan. To verify the RNA-Seq results, 6 candidate genes were randomly selected and evaluated by qRT-PCR) in this study. The results indicated that the expression levels of DEGs obtained by qRT-PCR were nearly consistent with those obtained by RNA-Seq, demonstrating the validity of the transcriptomic RNA-Seq data (**Figure 1A**).

DEGs analysis was carried out by comparing the gene transcriptional levels in myrislignan-treated *T. gondii* and that in untreated control *T. gondii*, the DEGs in each treatment group in **Table 1**. Venn diagram analysis revealed 63 genes that were differentially co-expressed in myrislignan treated groups (32, 50 or 70 μg/mL) (**Figure 1B**). GO enrichment analysis of DEGs revealed changes in biological processes, molecular functions and cellular components in *T. gondii* after treatment with myrislignan (**Figure 1C**), and the most common GO terms in these categories were “catalytic activity”, such as “oxidoreductase activity”, “oxidation−reduction process” and “electron transfer activity”. KEGG pathway analysis showed that the DEGs were mainly associated with “oxidative phosphorylation”, as shown in **Figure 1D**.

**Table 1:** Statistics of number of differentially expressed genes (DEGs).

Compare_group	up	down	total
Control -vs- MyrH	836	138	974
Control -vs- MyrM	309	57	366
Control -vs- MyrL	76	107	183

MyrH (Myrislignan 70 μg/mL); MyrM (Myrislignan 50 μg/mL); MyrL (Myrislignan 32 μg/mL).

### Surface Plasmon Resonance (SPR) Analysis

**Figure 2:**
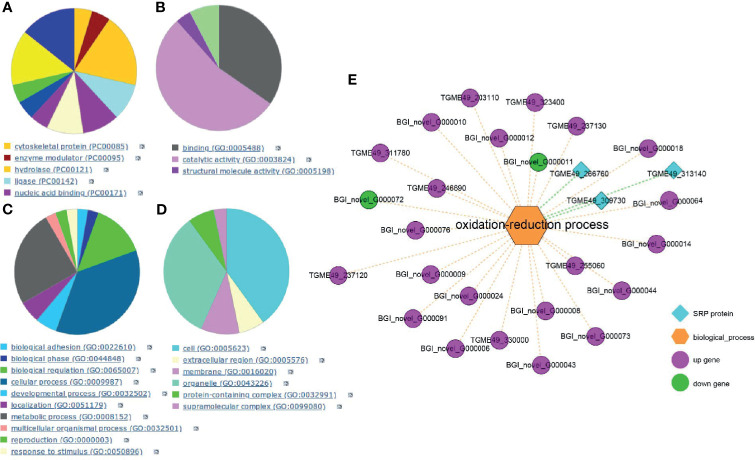
SPR analysis. GO database analysis of the affinity proteins classification in *T. gondii* **(A)**, including categories: molecular function classification **(B)**, biological process **(C)**; cell component **(D)**. Integration of transcriptomics and SPR for target discovery **(E)**.

To more precisely identify a target protein, SPR analysis investigated the affinity and interactive effect between myrislignan and the *T. gondii* proteins. A total of 58 *T. gondii* proteins were captured by myrislignan (**Supplementary Dataset S2**). The kinetic affinity between *T. gondii* peptides and myrislignan was calculated. Accordingly, 26 specific binding proteins indicating high affinity with binding scores of greater than 1,000, were selected for the following experiments. According to Gene Ontology (GO) database, the target proteins were further analysed by functional clustering and enrichment. The results of protein classification were shown in **Figure 2A**, the results of molecular function classification were shown in **Figure 2B**, the biological process classification results were shown in **Figure 2C**, and the cell component classification of the results were shown in **Figure 2D**.

In order to verify and identify new mechanisms of action of myrislignan, RNA-Seq data and SPR-MS data (26 proteins) were compared and combined. We found the DEGs and high affinity proteins were enriched in the oxidation-reduction process (**Figure 2E**). Therefore, myrislignan may play a key role of anti-*T. gondii* activity by affecting the oxidation-reduction process of *T. gondii*.

### Myrislignan Induced the Production of SOD and ROS in *T. gondii* Tachyzoites

**Figure 3:**
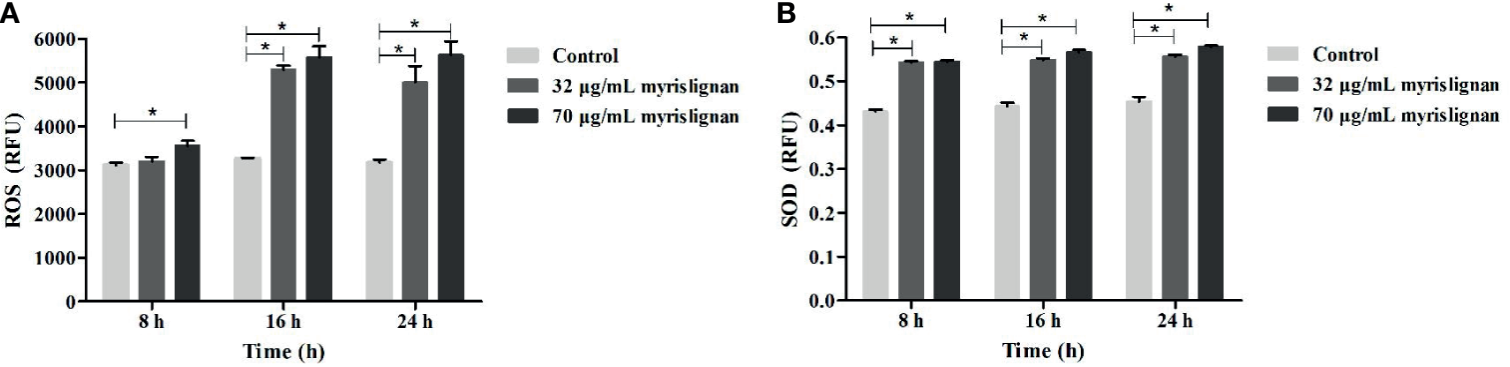
Myrislignan destroyed the oxidation-reduction in *T. gondii* Tachyzoites. Myrislignan induced the increased in ROS of *T. gondii* **(A)**. 1×10^6^ tachyzoites were treated with myrislignan (32 or 70 μg/mL) in DMEM or with no drug (as control) for 8 h, 16 h or 24 h, then incubated with 10 µM DCFH-DA probe for 20 min at 37°C, and the luminescence of each sample was detected using a multilabel reader. Myrislignan upregulated SOD activity in *T. gondii* **(B)**, Tachyzoites (1×10^6^ per group) were lysed after incubation with myrislignan (32 or 70 μg/mL) or without drug (control), the supernatant was added to a 96 well plates, and the absorbance of each sample was measured at 450 nm by SOD assay kit. *p < 0.01 compared with the parasite control.

We investigated whether myrislignan stimulated the increase of ROS production in *T. gondii* tachyzoites. After myrislignan incubation, ROS activity was also significantly (*p*<0.01) upregulated (**Figure 3A**). SOD is an important antioxidant produced by parasitic protozoa. It can maintain the stability of the internal environment and prevent the clearance of host immune cells. Therefore, we evaluated the SOD activity of *T. gondii* RH tachyzoites and found that the content of SOD was increased after myrislignan incubation, but did not increase over time (**Figure 3B**).

### Myrislignan Induced Autophagy in *T. gondii*

**Figure 4:**
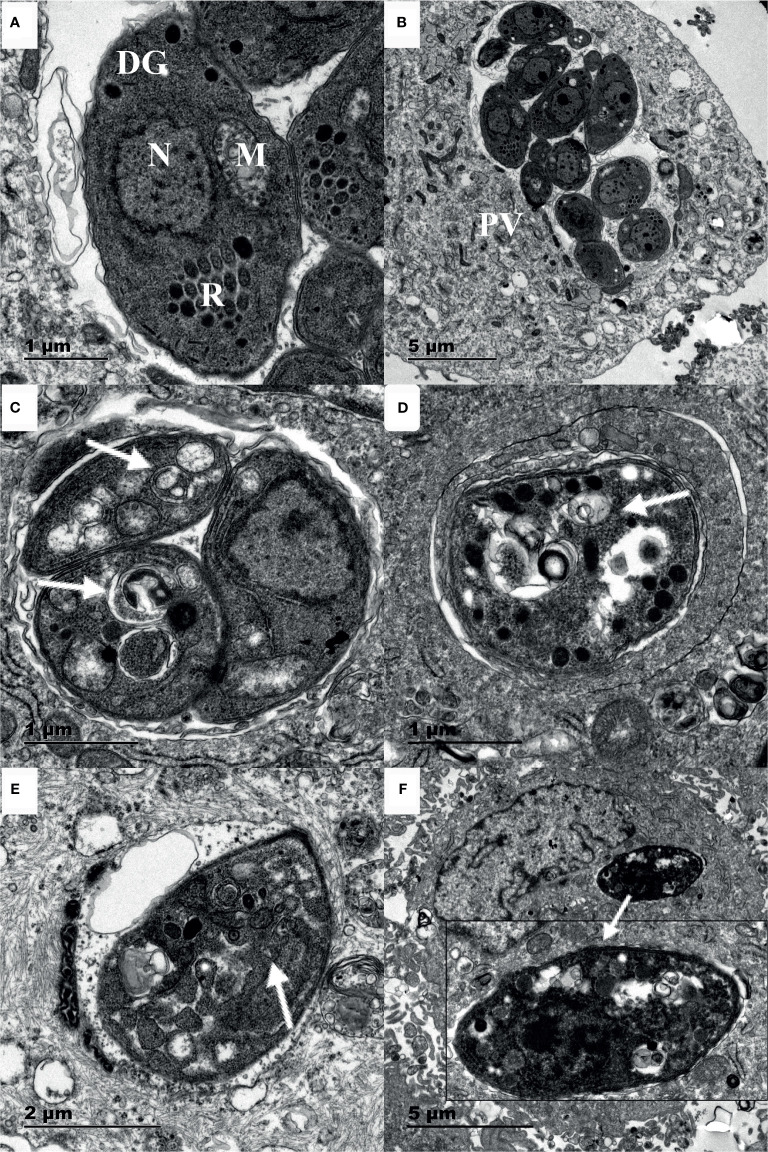
Ultrastructural changes in *T. gondii* tachyzoites after myrislignan. The well-preserved tachyzoite structures were maintained in the control group, including the nucleus (N), rhoptries (R), dense granules (DGs) and mitochondrion (M) **(A, B)**. Myrislignan treatment for 16 h caused many autophagic vacuoles to emerge in the cytoplasm **(C, E)**, as indicated by the arrows. After myrislignan treatment for 24 h, the cytoplasmic structure and parasitophorous vacuole (PV) membranes of tachyzoites had completely disappeared **(F)**. Scale bars: 1 μm **(A, C, D)**; 2 μm **(E)**; 5 μm **(B, F)**.

Myrislignan treatment for 16 h caused many autophagic vacuoles to emerge in the cytoplasm, as indicated by the arrows; all of these effects are hallmarks of autophagy (**Figures 4C, E**). In addition, after 24 h of treatment with myrislignan, the cytoplasmic structure and parasitophorous vacuole (PV) membranes of tachyzoites had completely disappeared, and progressive degeneration of the parasites was observed, as shown in (**Figures 4D, F**). However, autophagic vacuoles were not frequently discovered in untreated *T. gondii*. The TEM results confirmed that the untreated parasites displayed a well-preserved intracellular space with typical apicomplexan structural features, including rhoptries (R), dense granules (DGs), a nucleus (N), and a mitochondrion (M) (**Figures 4A, B**). However, confirmation of these structures as autophagosomes will require the generation of specific markers.

**Figure 5:**
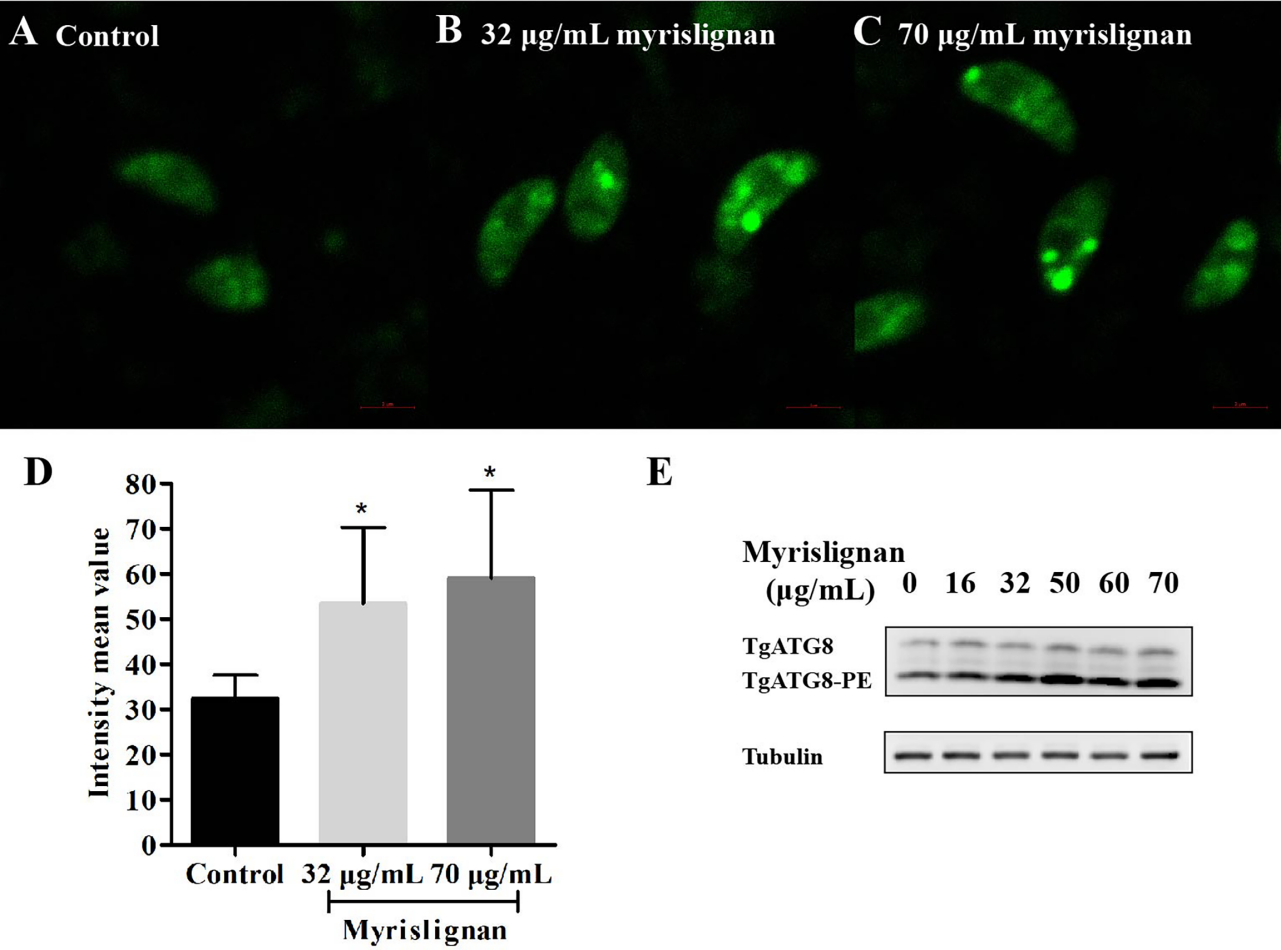
Myrislignan Induced autophagy in *T. gondii*. Myrislignan treatment induced distinct fluorescent dot-like structures corresponding to numerous autophagic vacuoles in *T. gondii* **(B, C)**, while no fluorescent dot-like structures were discovered in the untreated *T. gondii* **(A)**, the fluorescence intensity mean value of each group was shown in **(D)**, *p < 0.01 compared with the parasite control. Western blotting analysis showed that TgATG8-PE was upregulated after myrislignan treatment in a dose-dependent manner **(E)**.

To further confirm autophagy in *T. gondii* stimulated by myrislignan, MDC staining was exploited to detect numerous autophagic vacuoles in *T. gondii* after incubation with myrislignan. In MDC staining, myrislignan treatment resulted in obvious fluorescent spot-like structure of *T. gondii*, indicating a large number of autophagic vacuoles (**Figures 5B, C**), while there were no fluorescent dot-like structures in the untreated *T. gondii* (**Figure 5A**), the fluorescence intensity mean value of each group was shown in **Figure 5D**.

Furthermore, the expression of the typical autophagic marker TgATG8-PE was assessed in *T. gondii* in the absence or presence of myrislignan by western blotting analysis. As expected, the results showed that the autophagy marker TgATG8-PE was upregulated after myrislignan treatment in a dose-dependent manner (**Figure 5E**).

### Myrislignan Induced Cell Death of Tachyzoites

**Figure 6:**
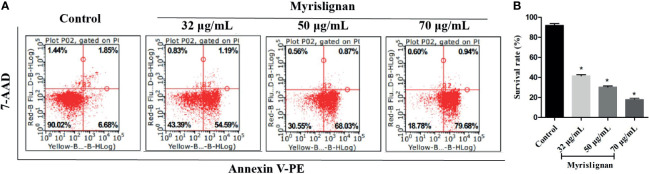
Myrislignan induced cell death of tachyzoites. Myrislignan (32, 50 and 70 μg/mL) treatment induced a decline in the *T. gondii* survival rates of approximately 42.02% ± 1.29%, 26.83% ± 3.29% and 17.22 ± 1.37%, respectively. The survival rate in the control group was approximately 88.53% ± 1.31%. The representative figure is shown in **(A)**. The proportions of *T. gondii* surviving after treatment with myrislignan (32, 50 or 70 μg/mL) for 24 h are shown in **(B)**. *p < 0.01 compared with the parasite control.

Furthermore, the cell death of inhibiting extracellular growing tachyzoites after treatment with different concentrations of myrislignan for 24 h was determined by flow cytometry (**Figure 6A**). The different patterns in the Annexin V-PE/7-AAD analysis were used to identify the different *T. gondii* populations where 7-AAD-negative and Annexin V-PE-negative cells were designated as viable tachyzoites. The proportion of viable tachyzoites varied from 88.53% ± 1.31% in control groups to 42.02% ± 1.29%, 26.83% ± 3.29%, 17.22 ± 1.37% in 32, 50 or 70 μg/mL myrislignan treatment groups, respectively. The results showed that myrislignan treatment had a concentration-dependent significant (*p < 0.01*) increase in cell death effect of *T. gondii* tachyzoites (**Figure 6B**).

## Discussion

*T. gondii* is an obligate intracellular pathogen that can infect almost all warm-blooded animals and humans (Ling et al., 2006), causing major health problems. In recent years, people are looking for safe and effective anti-*T. gondii* drugs. Natural products in plants have become an important source of clinical drugs, and some of the new compounds are expected to be the leaders of new drugs (Sepulveda-Arias et al., 2014). Myrislignan is a main active ingredient of nutmeg exhibiting various bioactivities, such as inducing apoptosis and cell cycle arrest in A549 cells (Lu et al., 2017), activating the AMPK enzyme and exerting anti-obesity effect (Nguyen et al., 2010), inhibiting the activation of NF-kB signalling pathway, reducing the inflammatory response of macrophages induced by lipopolysaccharide, and protecting the liver from thioacetamide injury (Jin et al., 2012; Yang et al., 2018). In previous study, myrislignan exerted the anti-*T. gondii* activity by inhibiting its replication and invasion *in vitro*, and reduces the parasite burden in the tissues of infected mice. Our previous findings suggested myrislignan against *T. gondii* might be associated with *T. gondii* mitochondrial function (Zhang et al., 2019). In this study, we also explore the action mechanism of myrislignan against *T. gondii*, and it will provide ideas for the development of new types of anti-*T. gondii* compounds, and contribute to the structural modification and optimization of myrislignan. In agreement with previous work, deep-sequencing analysis of RNAs of *T. gondii* after incubated with myrislignan in different concentrations revealed significant changes in “oxidoreductase activity” and “electron transfer activity” of “catalytic activity” in the DEGs of *T. gondii* by GO enrichment analysis. KEGG pathway analysis showed that the DEGs were mainly associated with “oxidative phosphorylation”. Furthermore, combined with SPR analysis, the DEG and high affinity proteins were enriched in the oxidation-reduction process, these indicated that myrislignan may have an anti-*T. gondii* activity by affecting the oxidation-reduction process of *T. gondii*.

In order to verify whether the anti-*Toxoplasma* effect of myrislignan is related to oxidation-reduction process of *T. gondii*, we examined the content of ROS and SOD. ROS is a by-product of aerobic metabolism, including superoxide anion, hydrogen peroxide and hydroxyl radical, which plays an important role in many biological processes (Schieber and Chandel, 2014). SOD is an important reductase widely existing in cells. It can promote the transformation of superoxide anion (O^2-^) into hydrogen peroxide and oxygen, and is one of the most important antioxidants for parasite protozoa to maintain homeostasis (Miller, 2012; Wang et al., 2018). In *T. gondii*, SOD can not only protect *T. gondii* from oxidative damage, but also participate in the growth process of tachyzoite (Odberg-Ferragut et al., 2000). We investigated that myrislignan strikingly increased the ROS content in tachyzoites with time-dependent manner in 24 h of incubation, indicating the instability of the intracellular redox balance by myrislignan. Furthermore, the SOD activity of RH tachyzoites incubated with myrislignan and found that RH tachyzoites maintained survival by upregulating SOD activity, but did not increase as time. However, the ROS activity induced by myrislignan increased significantly in a time-dependent manner. Therefore, we conceived of the idea that myrislignan may destroy the physiological redox biological signal, thus interfering with the metabolism or proliferation of parasites. Taken together, the significant increase of ROS activity and the abnormal production of SOD indicated that tachyzoites were in an environment of imbalanced internal redox system caused by myrislignan, thus gradually inhibiting the growth of extracellular tachyzoites.

According to our previous study, myrislignan against *T. gondii* might affect *T. gondii* mitochondrial function (Zhang et al., 2019). Mitochondria are not only the main site of ROS production, but also the main target of oxidative damage. Herein, we also indicated myrislignan increased the ROS, destroyed the oxidant-antioxidant homeostasis of tachyzoites, then led to oxidative stress. Taken together, we infer that myrislignan may reduce the mitochondrial membrane potential and ATP level of *T. gondii* and damage mitochondrial function by interfering the redox- antioxidant process of *T. gondii*. In addition, *T. gondii* is different from mammals, it has only one mitochondrion (Melo et al., 2000). The mitochondrial damage may lead to autophagy. Furthermore, in order to explore the effect of redox injury on *T. gondii*, TEM analysis confirmed the presence of autophagy-like structures. Autophagosome is formed by cup-shaped single membrane structure, also known as separation membrane or pre-autophagosome. The maturation of this structure is the conversion of the ATG8 from a diffuse cytosolic form (ATG8) to a lapidated form (ATG8-PE), which associates with the isolated membrane and specially localizes on the inner autophagosome membrane. Thus, ATG8 is a widely used marker for autophagy (Ghosh et al., 2012; Besteiro, 2017). Therefore, we detect the accumulation of TgATG8-PE by western blot analysis on the autophagy of *T. gondii* after incubation with myrislignan (Besteiro et al., 2011; Gao et al., 2014). Myrislignan caused a dose-dependent increase in TgATG8-PE protein levels in *T. gondii*, indicating activation of autophagy. To further confirm that myrislignan induced autophagy, MDC staining was used to stain myrislignan treated *T. gondii*. Abundant autophagic vacuoles appeared in the cytoplasm of *T. gondii*. Recently, some compounds have been found to cause *T. gondii* death by activating autophagy, such as monensin, the data indicated that autophagy as a potentially important mode of cell death of protozoan parasites in response to drugs (Lavine and Arrizabalaga, 2012). Moreover, myrislignan induced death in *T. gondii* by flow cytometric assessment. Given the results, myrislignan may induce autophagy by damaging the oxidation-reduction process, eventually leading to *T. gondii* death.

In conclusion, our results demonstrated that myrislignan can interfere with the redox homeostasis of the parasites, activate autophagy, and leading to *T. gondii* metabolic disorder and death, but the specific mechanism of action needs to explore.

## Data Availability Statement

The datasets presented in this study can be found in online repositories. The names of the repository/repositories and accession number(s) can be found below: NCBI PRJNA753595.

## Author Contributions

HS and JC revised the manuscript. JYZ directed the project. JLZ supervised the experiments and wrote the manuscript. BL, KL, BY, LG, XW, and CS reviewed the manuscript. All authors contributed to the article and approved the submitted version.

## Funding

This work was supported by Natural Science Fund of Gansu Provincial Science and Technology Project (20JR10RA023), the Science and Technology Project Fund of Gansu Province-Fundamental Research Innovative Groups (18JR3RA397) and the earmarked fund for the China Agriculture Research System (CARS-37).

## Conflict of Interest

The authors declare that the research was conducted in the absence of any commercial or financial relationships that could be construed as a potential conflict of interest.

## Publisher’s Note

All claims expressed in this article are solely those of the authors and do not necessarily represent those of their affiliated organizations, or those of the publisher, the editors and the reviewers. Any product that may be evaluated in this article, or claim that may be made by its manufacturer, is not guaranteed or endorsed by the publisher.

## Supplementary Material

Supplementary Data Sheet 1 The qRT-PCR primers used for RNA-seq validation.

Supplementary Data Sheet 2 The specific binding proteins of *T. gondii* were captured by myrislignan as determined using SPR.
