# Supplementary figures and images for: Myrislignan Induces Redox Imbalance and Activates Autophagy in Toxoplasma gondii
Source: Front Cell Infect Microbiol. 2021 Sep 3;11:730222. doi: 10.3389/fcimb.2021.730222 (PMC8447958; doi:10.3389/fcimb.2021.730222)

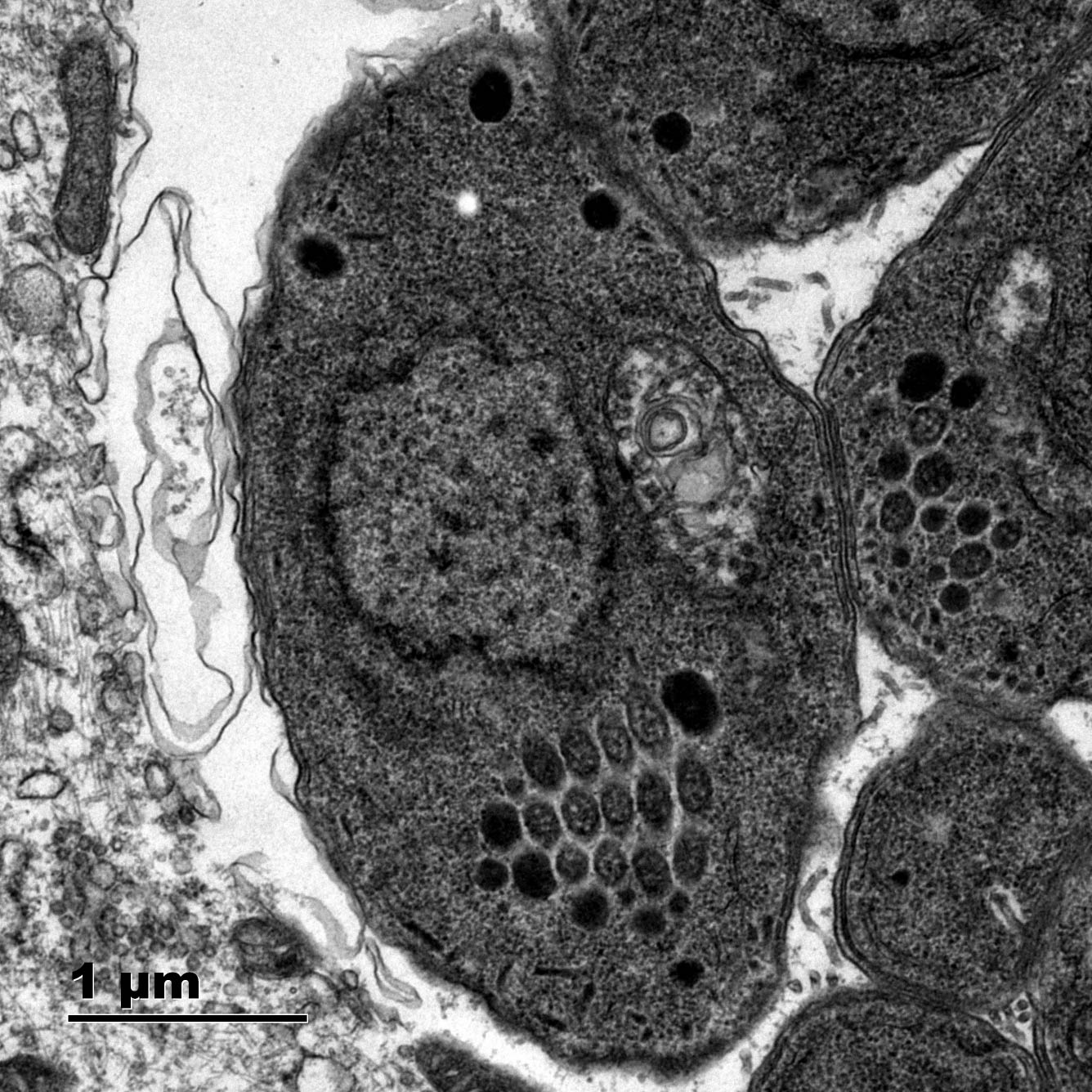

Supplement: Supplementary file 4 [file DataSheet_4.zip › Fig.4-raw data/A-Control-16 h_╗¡░σ 1.jpg]

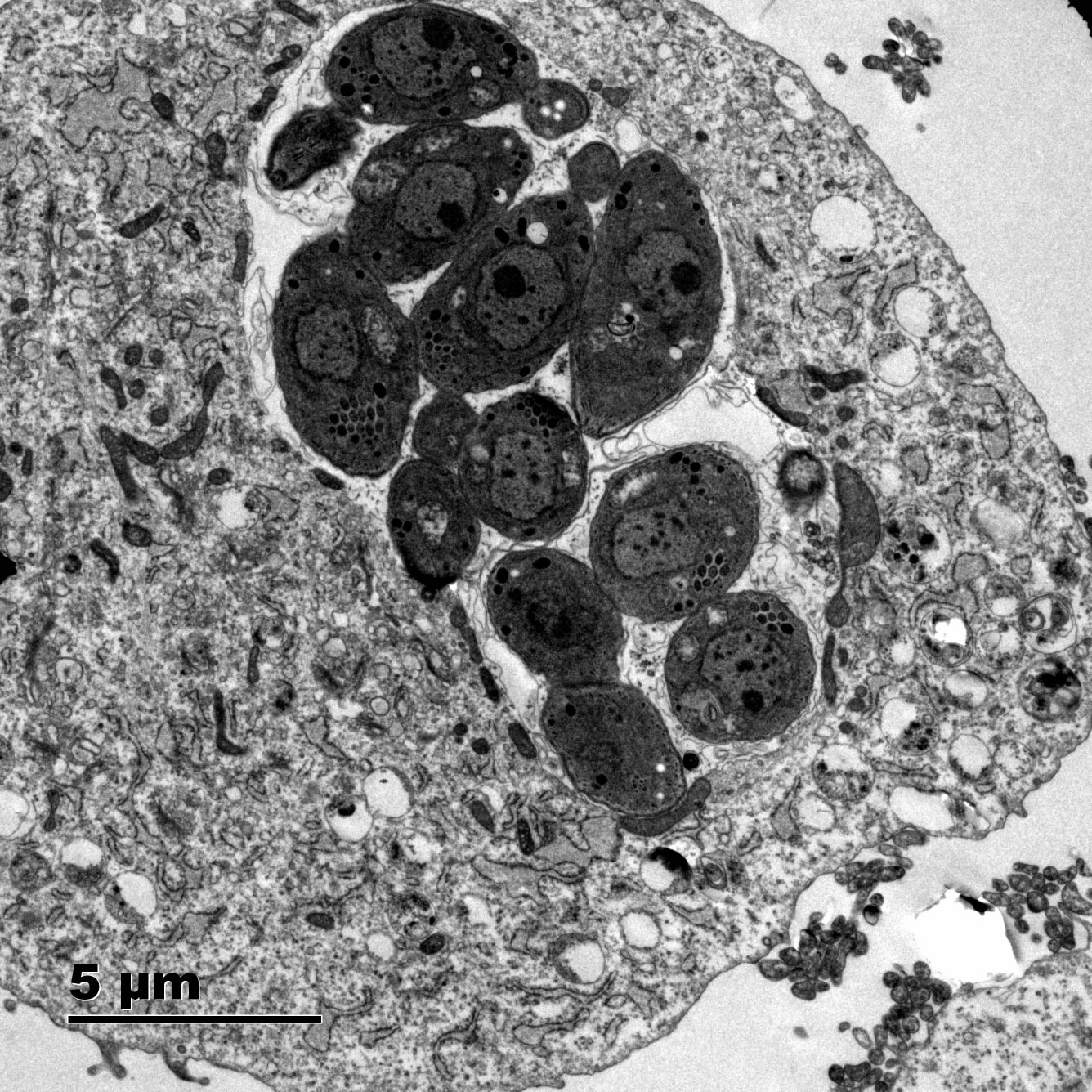

Supplement: Supplementary file 4 [file DataSheet_4.zip › Fig.4-raw data/B-Control-24 h_╗¡░σ 1.jpg]

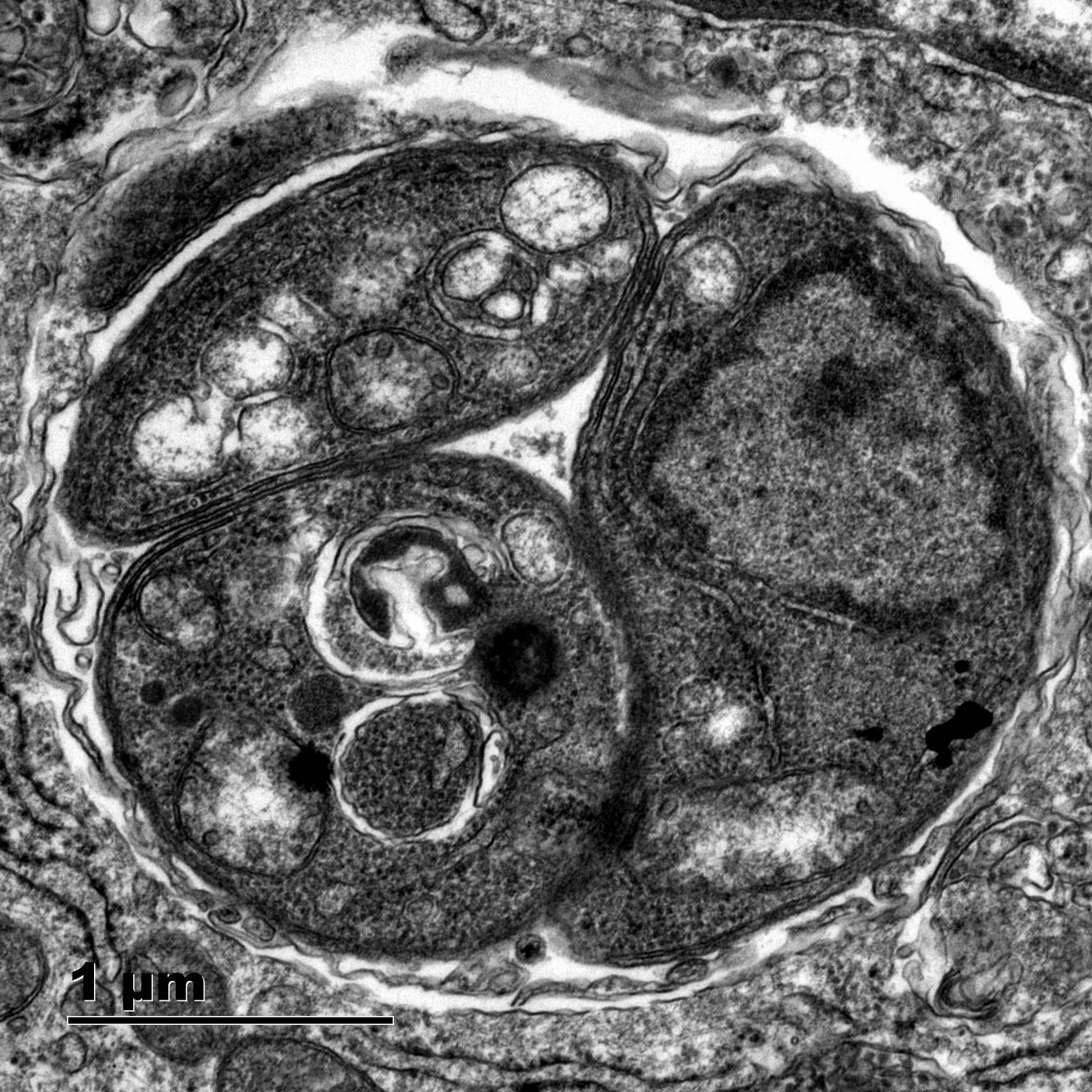

Supplement: Supplementary file 4 [file DataSheet_4.zip › Fig.4-raw data/C-32-myrislignan-16 h_╗¡░σ 1.jpg]

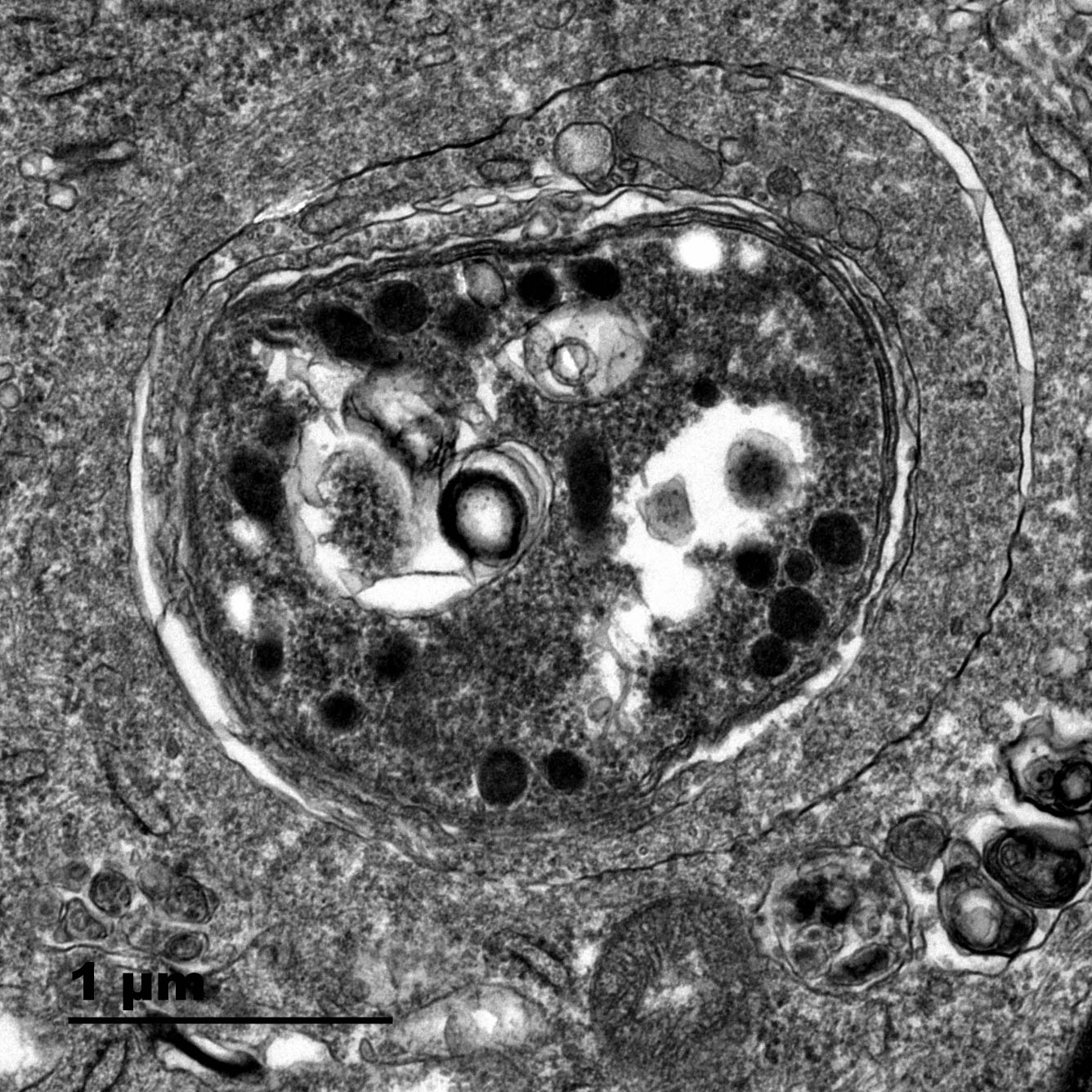

Supplement: Supplementary file 4 [file DataSheet_4.zip › Fig.4-raw data/D-32-myrislignan-24 h_╗¡░σ 1.jpg]

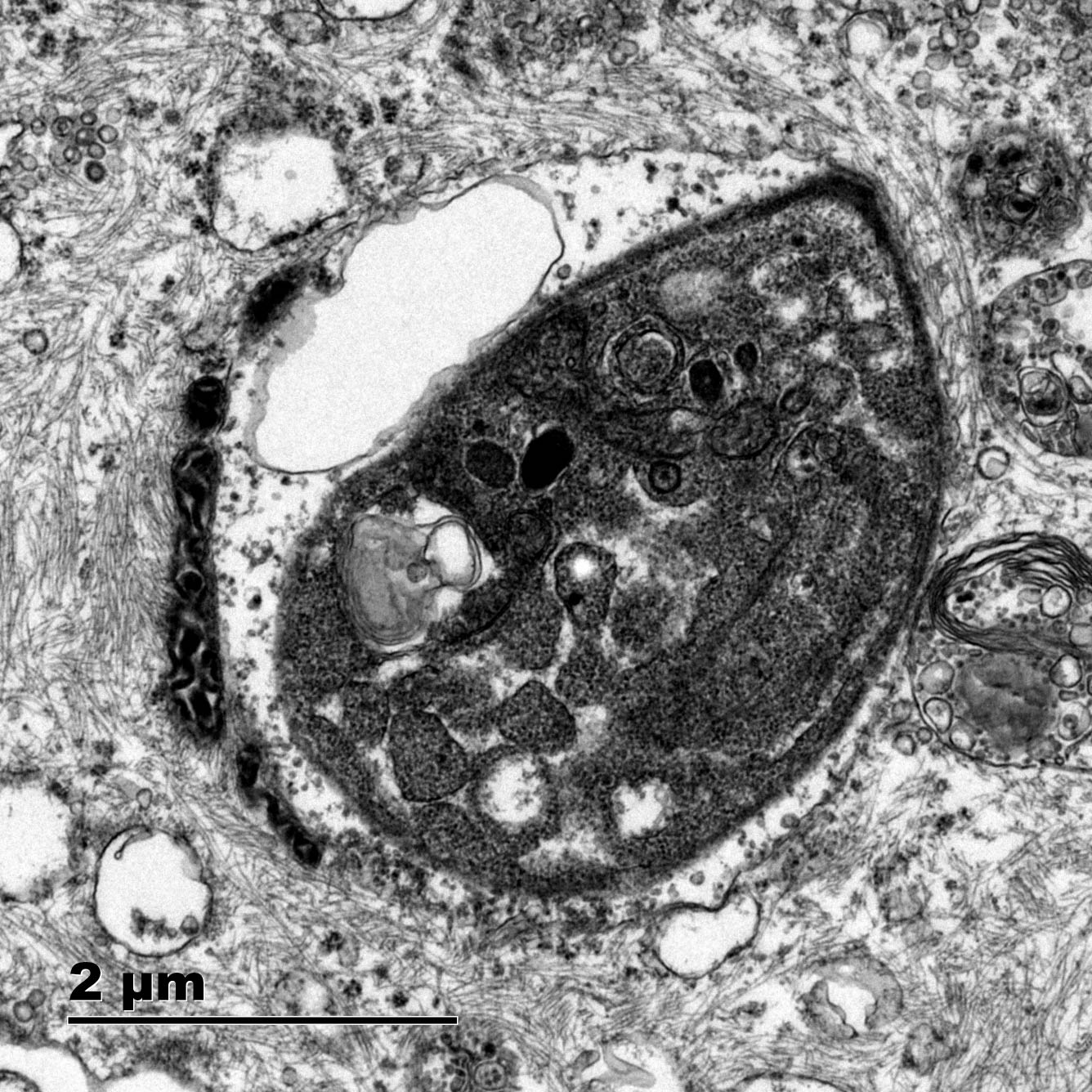

Supplement: Supplementary file 4 [file DataSheet_4.zip › Fig.4-raw data/E-70-myrislignan-16 h_╗¡░σ 1.jpg]

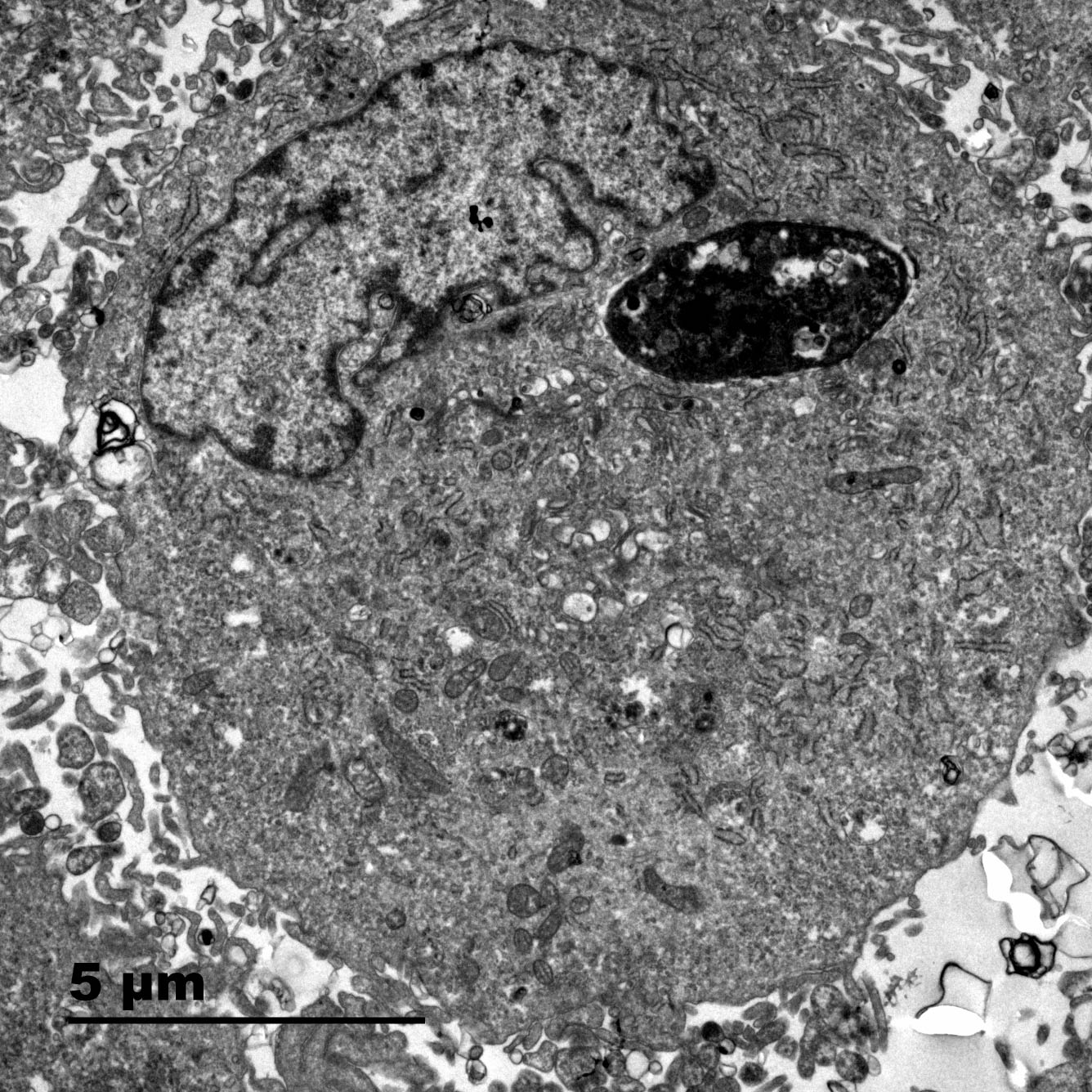

Supplement: Supplementary file 4 [file DataSheet_4.zip › Fig.4-raw data/F-70-myrislignan-24 h_╗¡░σ 1.jpg]

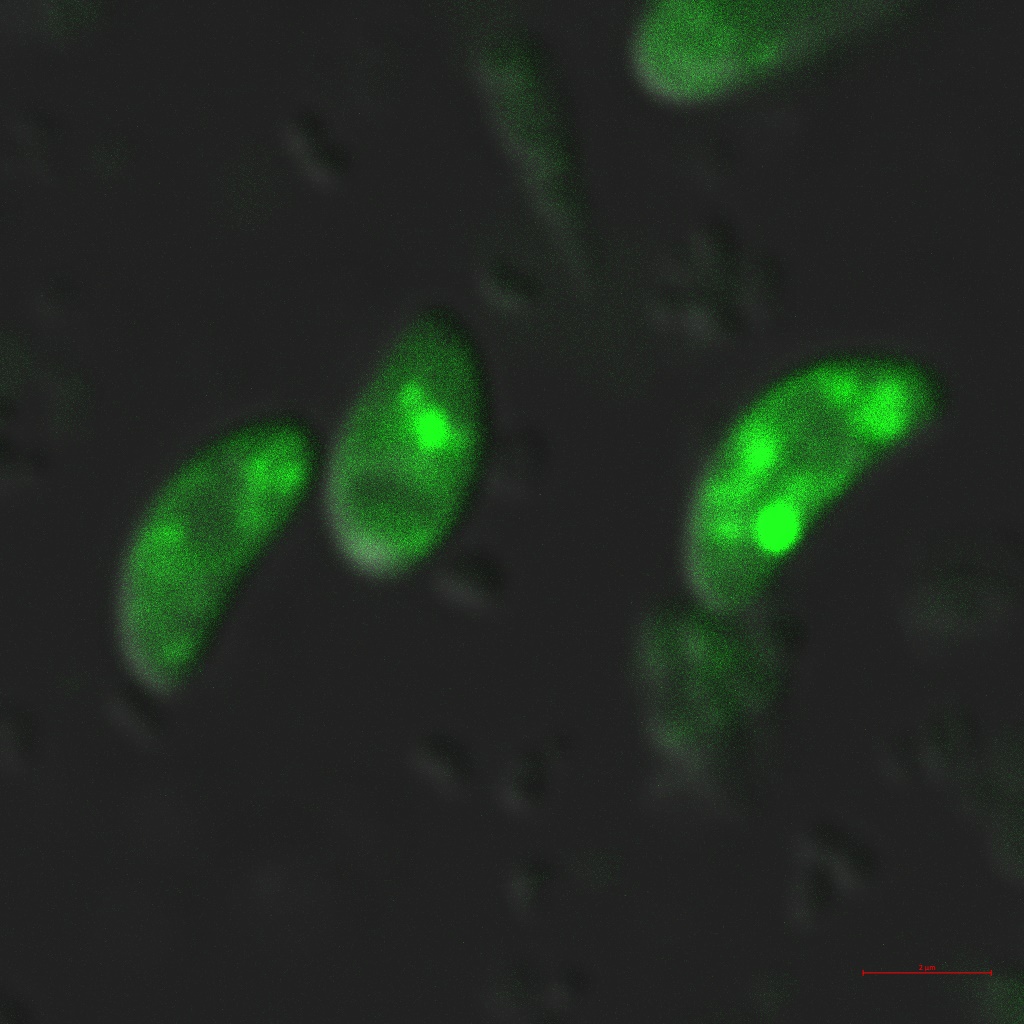

Supplement: Supplementary file 5 [file DataSheet_5.zip › Fig.5-raw data/32 a╠g/32-Myr_c1-2.jpg]

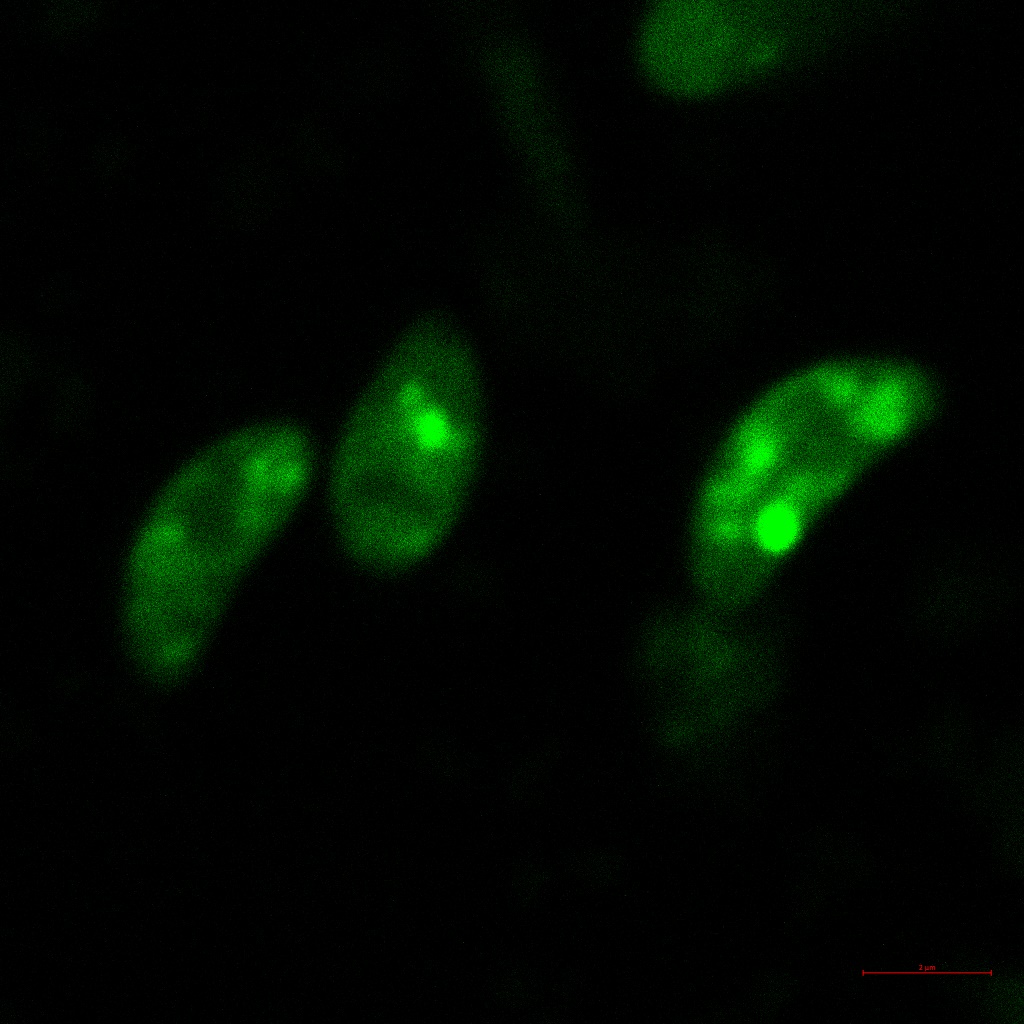

Supplement: Supplementary file 5 [file DataSheet_5.zip › Fig.5-raw data/32 a╠g/32-Myr_c1.jpg]

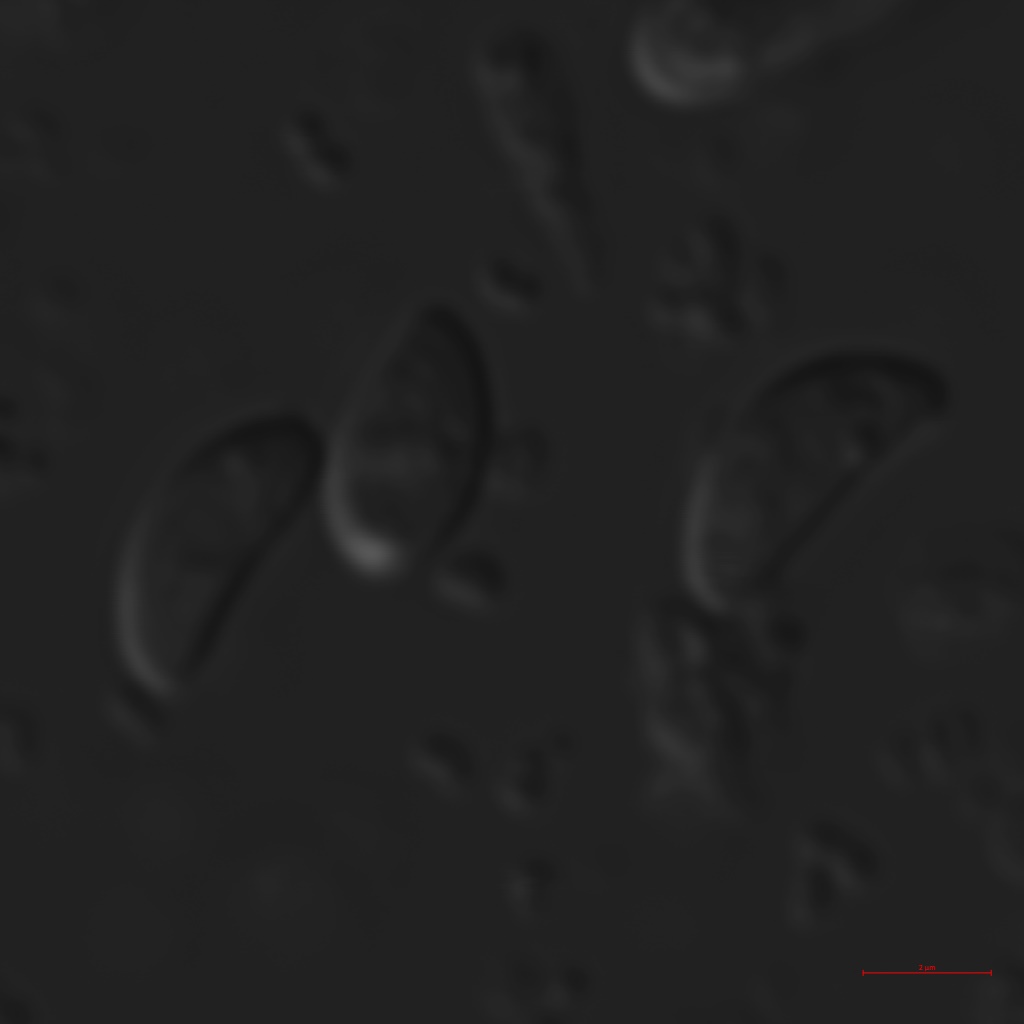

Supplement: Supplementary file 5 [file DataSheet_5.zip › Fig.5-raw data/32 a╠g/32-Myr_c2.jpg]

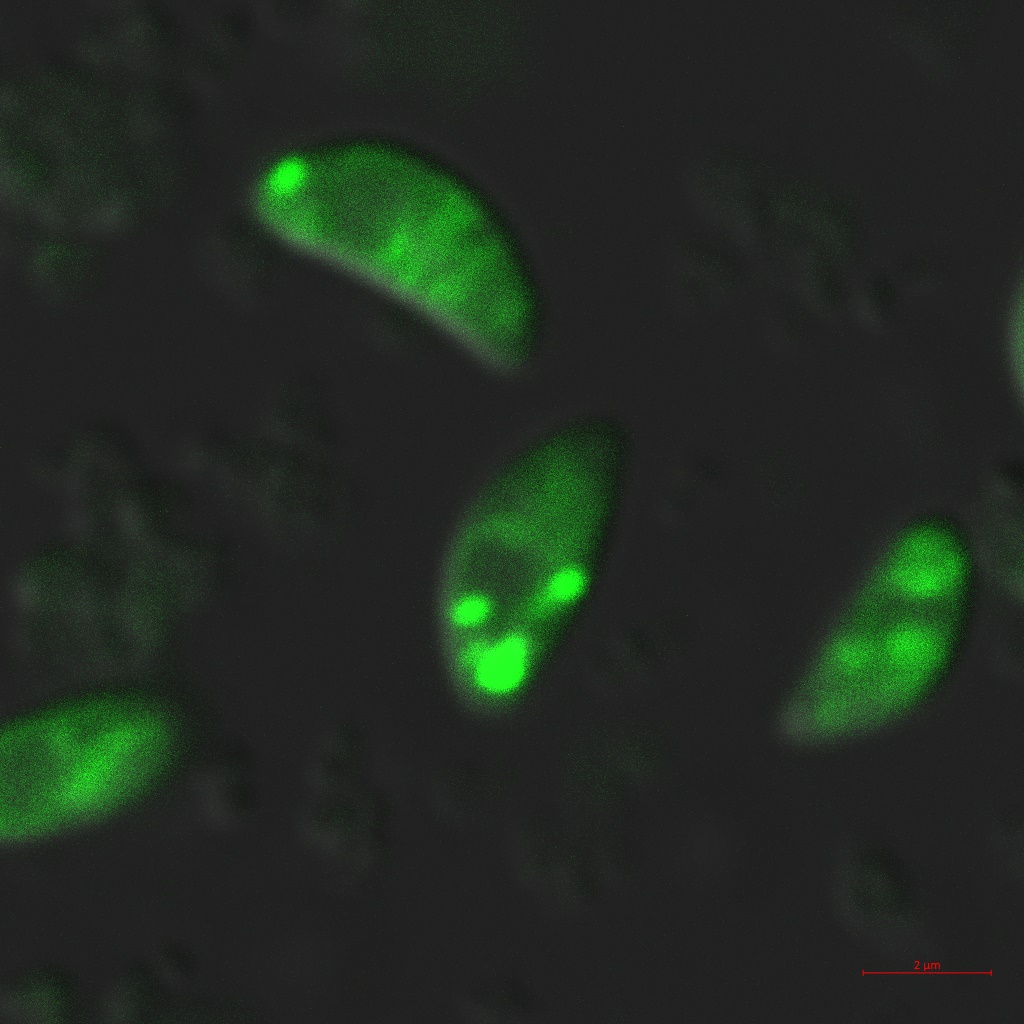

Supplement: Supplementary file 5 [file DataSheet_5.zip › Fig.5-raw data/70 a╠g/70-Myr_c1-2.jpg]

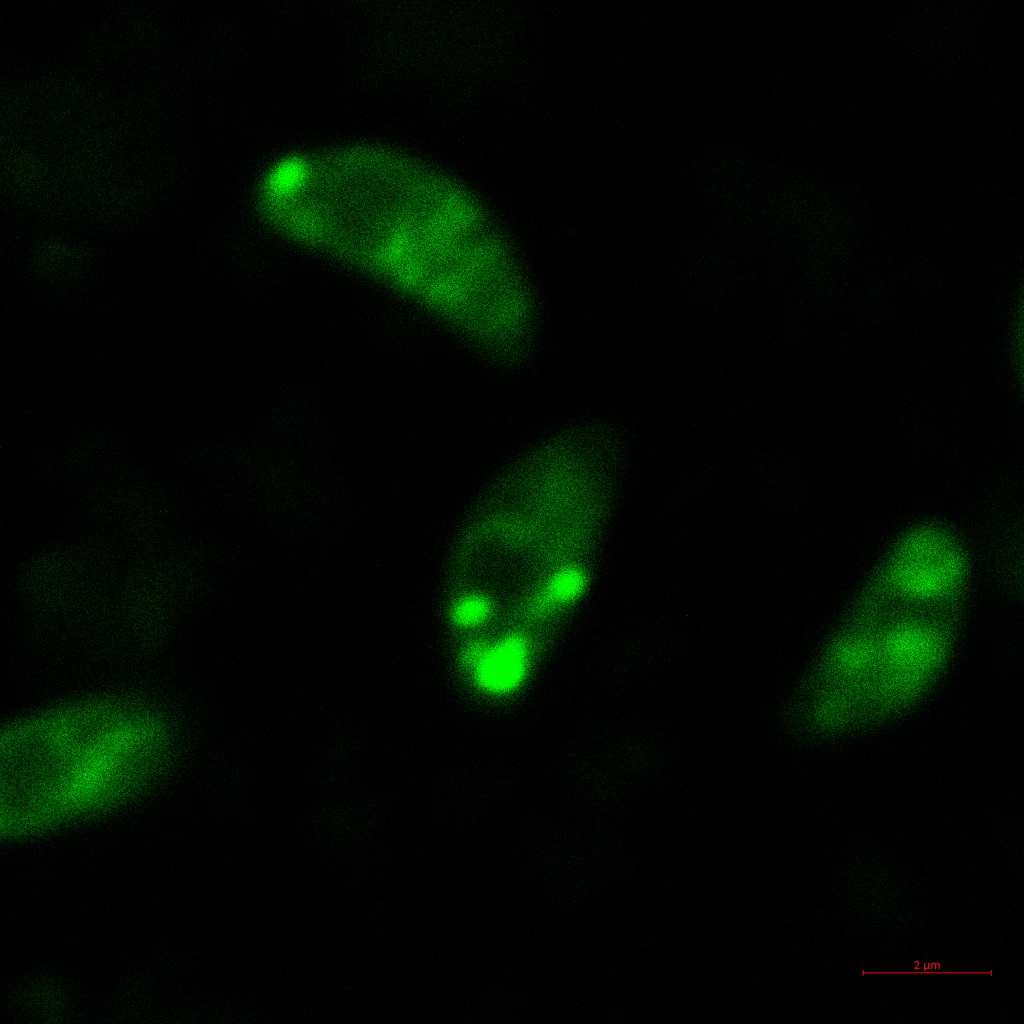

Supplement: Supplementary file 5 [file DataSheet_5.zip › Fig.5-raw data/70 a╠g/70-Myr_c1.jpg]

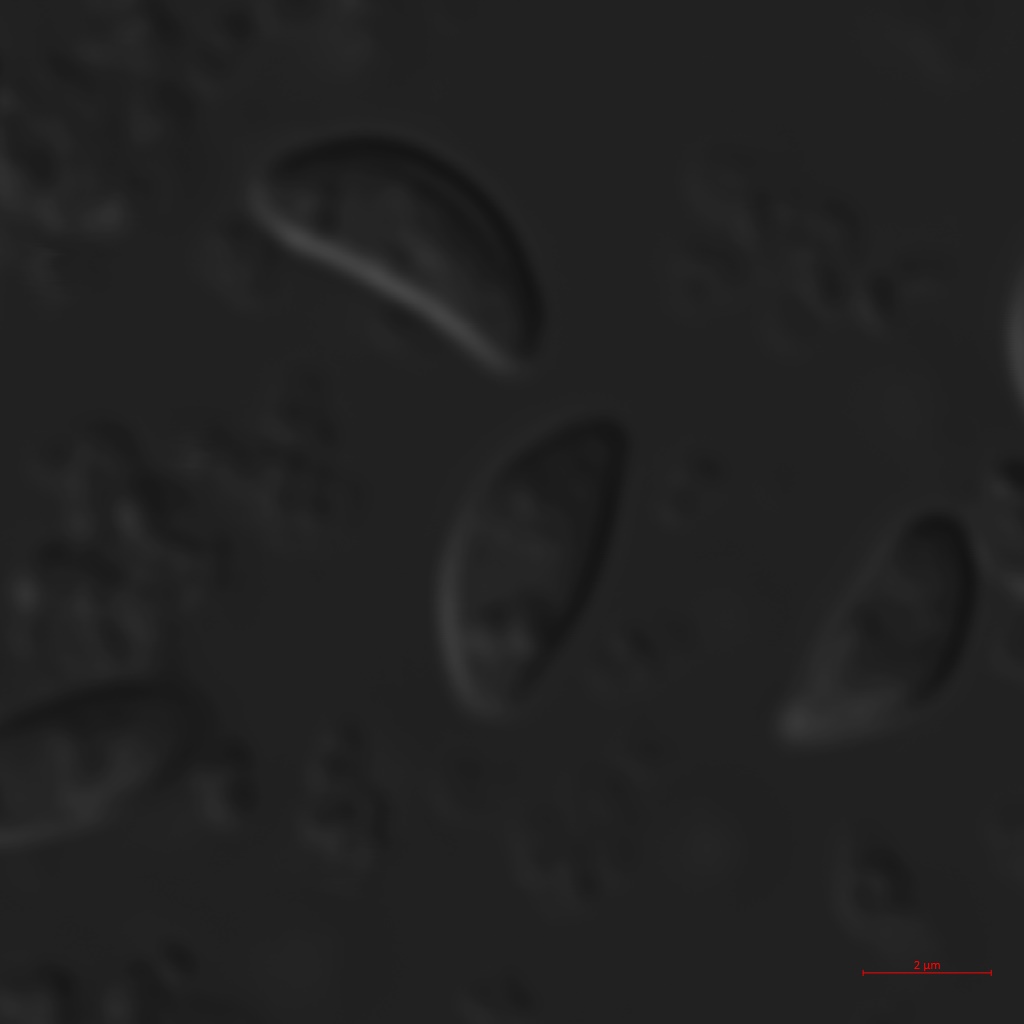

Supplement: Supplementary file 5 [file DataSheet_5.zip › Fig.5-raw data/70 a╠g/70-Myr_c2.jpg]

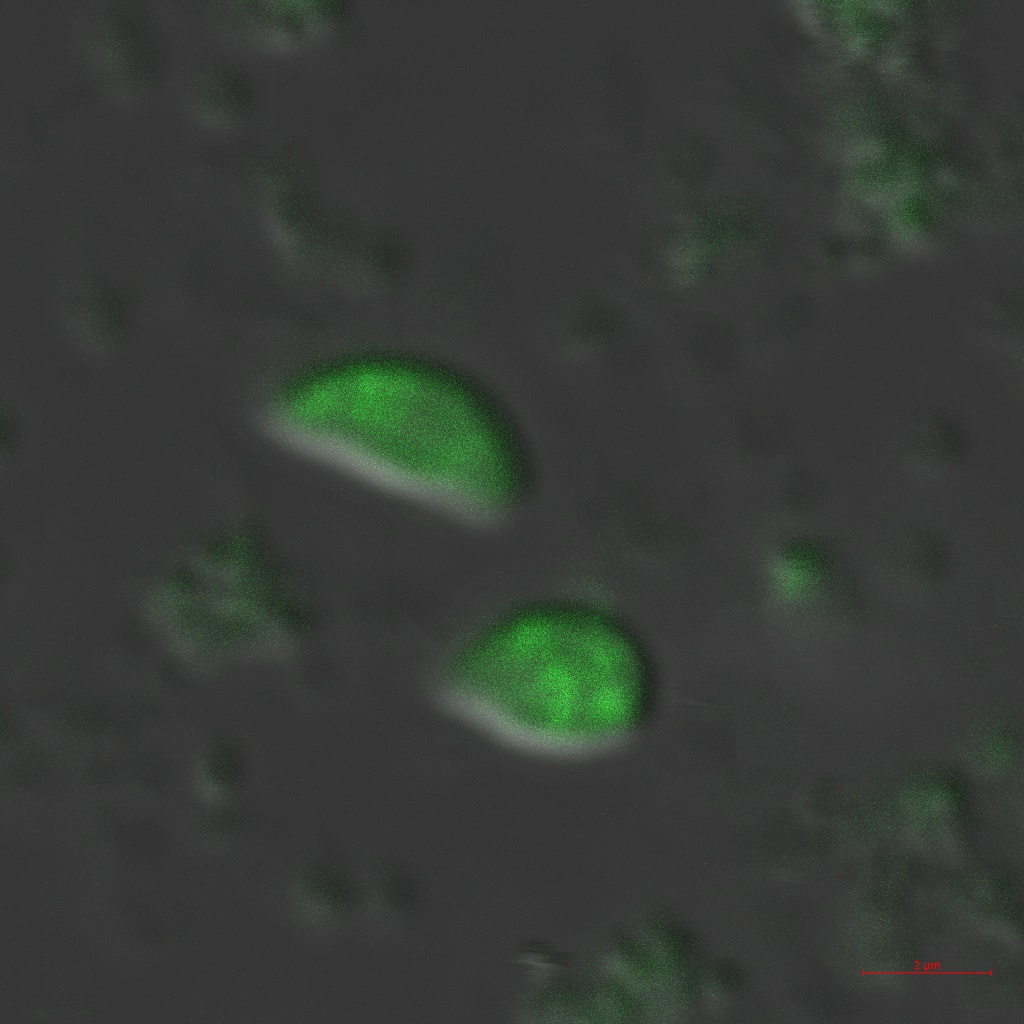

Supplement: Supplementary file 5 [file DataSheet_5.zip › Fig.5-raw data/Control/Control_c1-2.jpg]

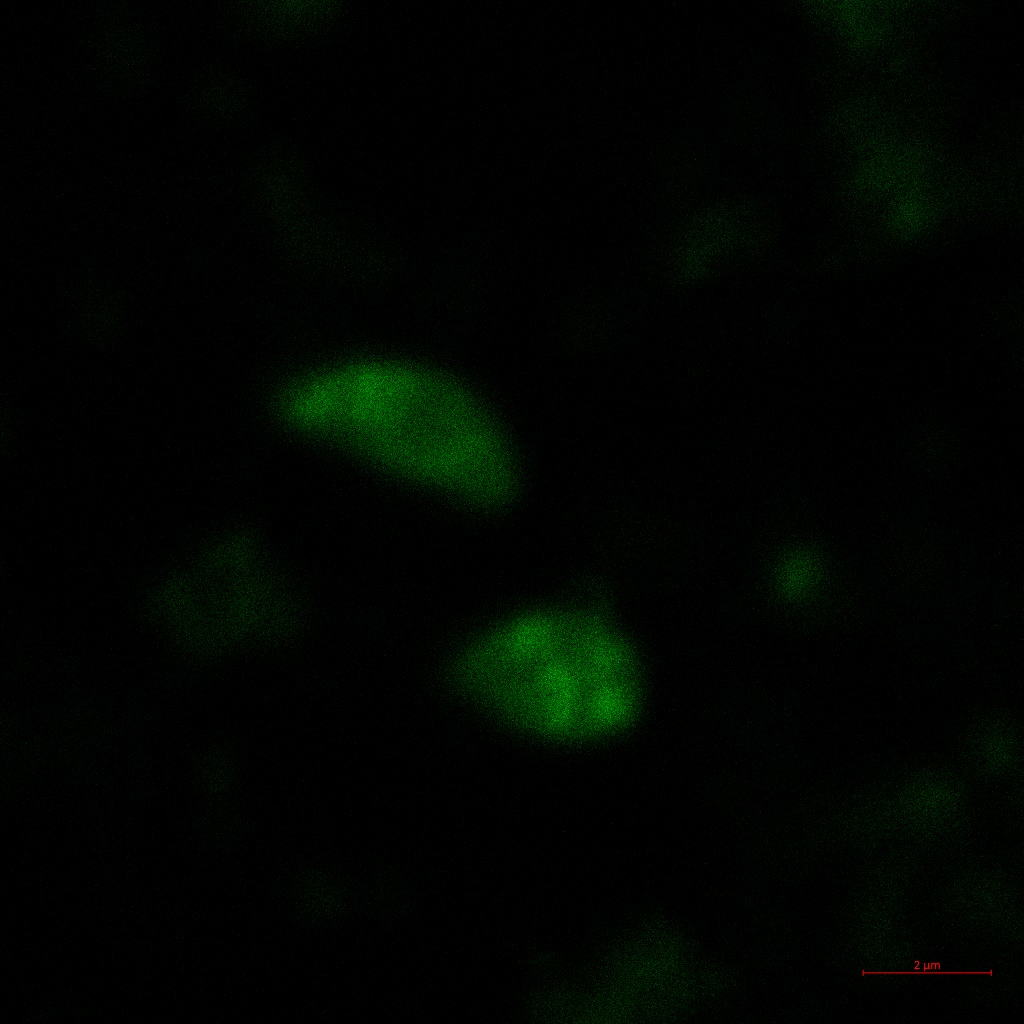

Supplement: Supplementary file 5 [file DataSheet_5.zip › Fig.5-raw data/Control/Control_c1.jpg]

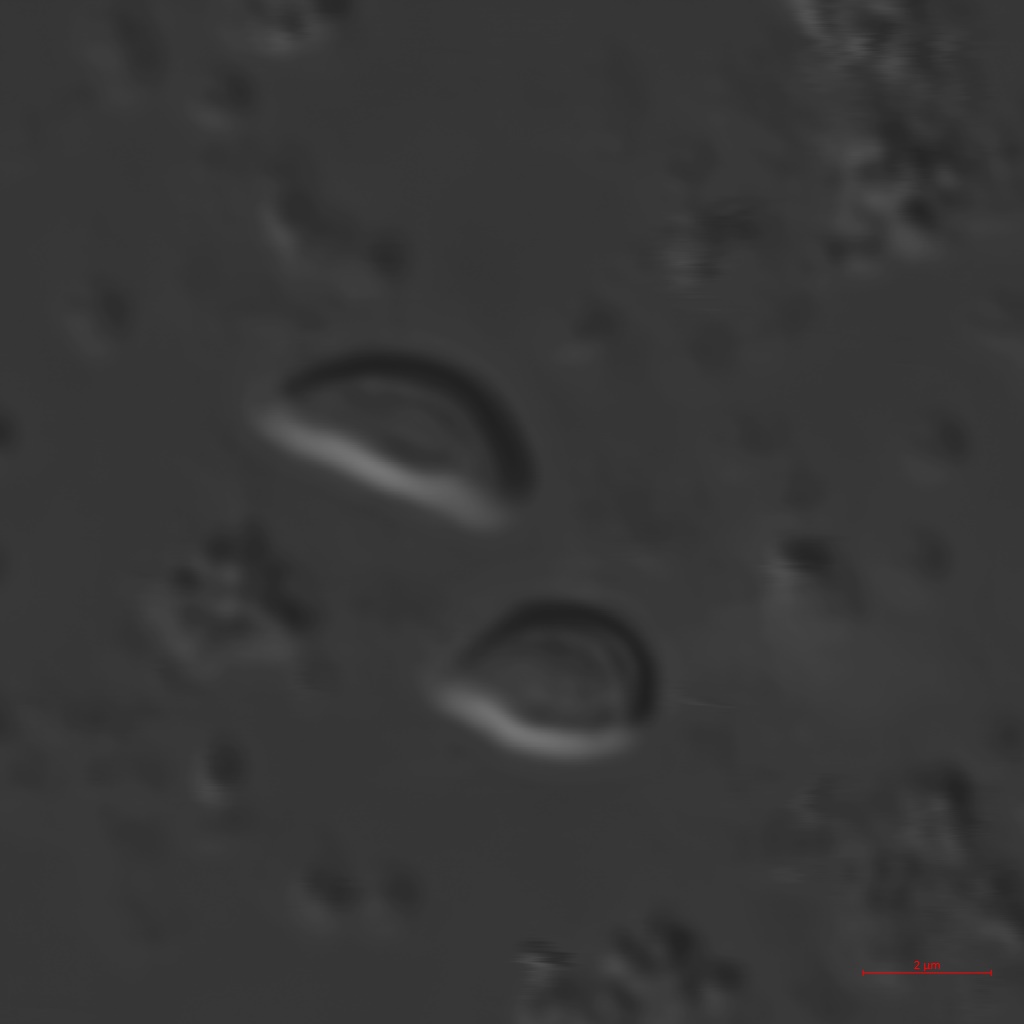

Supplement: Supplementary file 5 [file DataSheet_5.zip › Fig.5-raw data/Control/Control_c2.jpg]

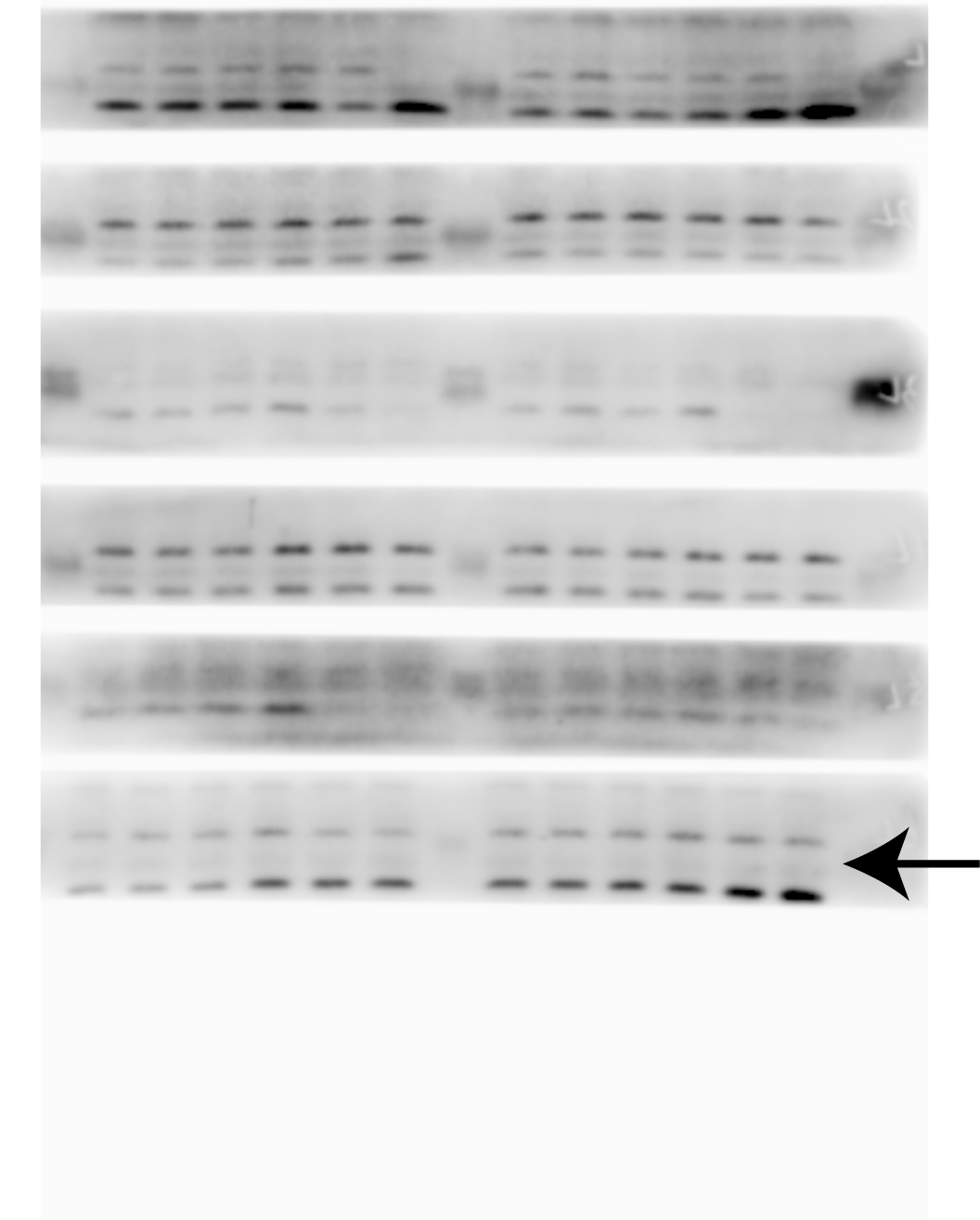

Supplement: Supplementary file 5 [file DataSheet_5.zip › Fig.5-raw data/WB╘¡╩╝═╝/LC3 2020.12.23_02.08.58-04_Chfb.tif]

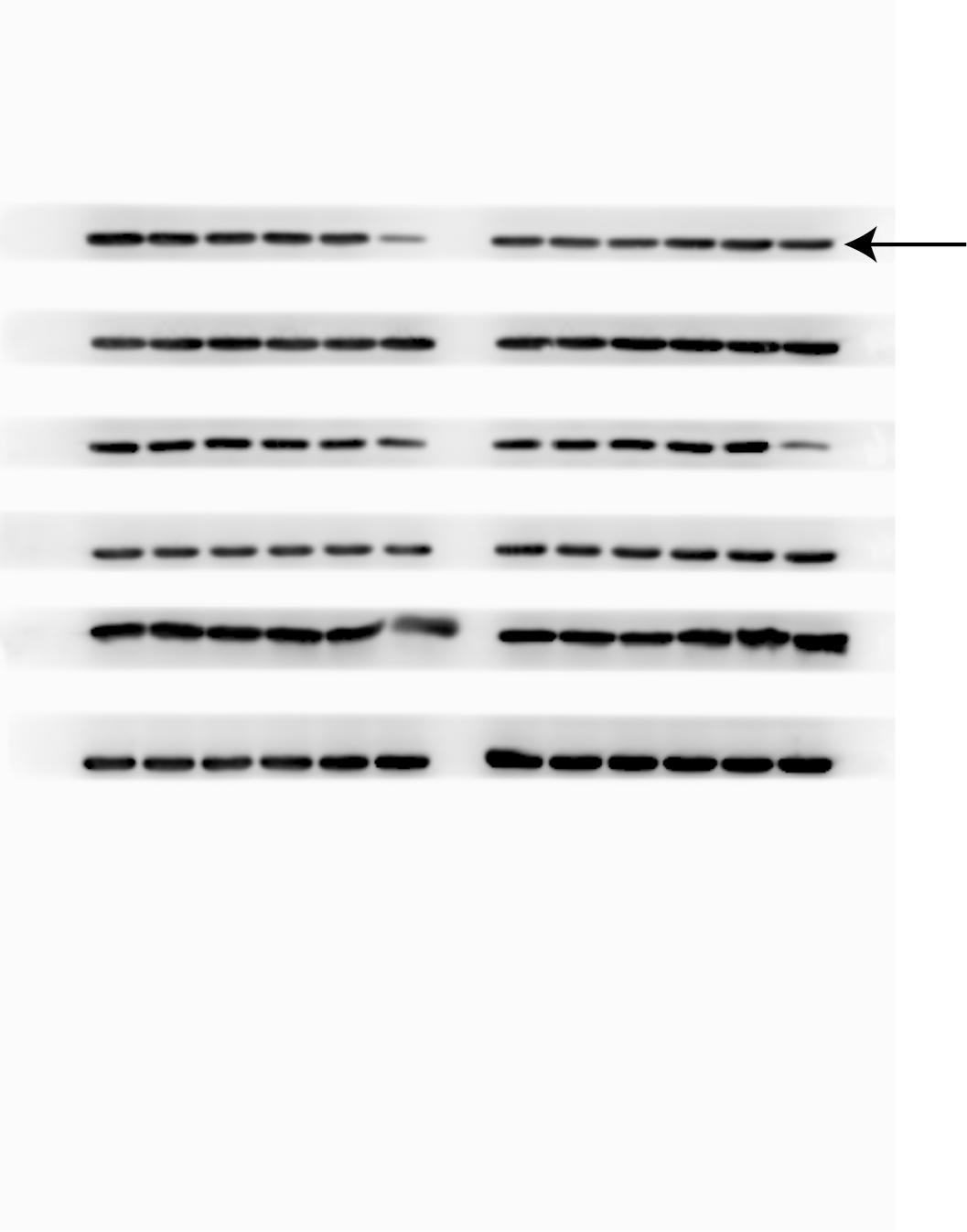

Supplement: Supplementary file 5 [file DataSheet_5.zip › Fig.5-raw data/WB╘¡╩╝═╝/Tubulin, 2020.12.23_01.29.45_Chfb.tif]
