# Supplementary material for: Myrislignan Induces Redox Imbalance and Activates Autophagy in Toxoplasma gondii
Source: Front Cell Infect Microbiol. 2021 Sep 3;11:730222. doi: 10.3389/fcimb.2021.730222 (PMC8447958; doi:10.3389/fcimb.2021.730222)
Supplement: Supplementary file 6 [file DataSheet_6.zip › Fig.6-raw data/2021-06-05_at_10-40-12pm-TOXOPLASMA-Myrislignan-1.pdf]

Well Number: A01 Sample ID: Annexin v

File Name: D:/shf/2021-06-05_at_10-40-12pm-TOXOPLASMA-Myrislignan-apptosis.fcs

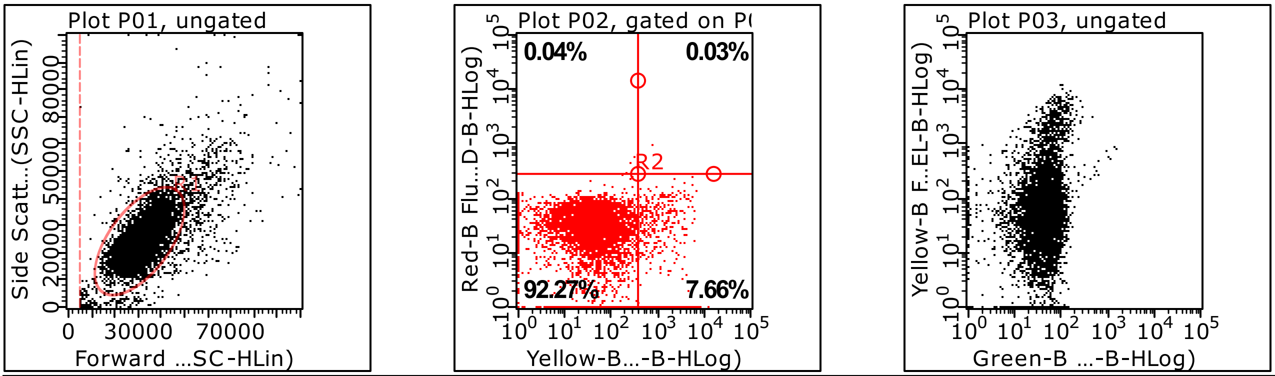

Well Number: A02 Sample ID: 7AAD

File Name: D:/shf/2021-06-05_at_10-40-12pm-TOXOPLASMA-Myrislignan-apptosis.fcs

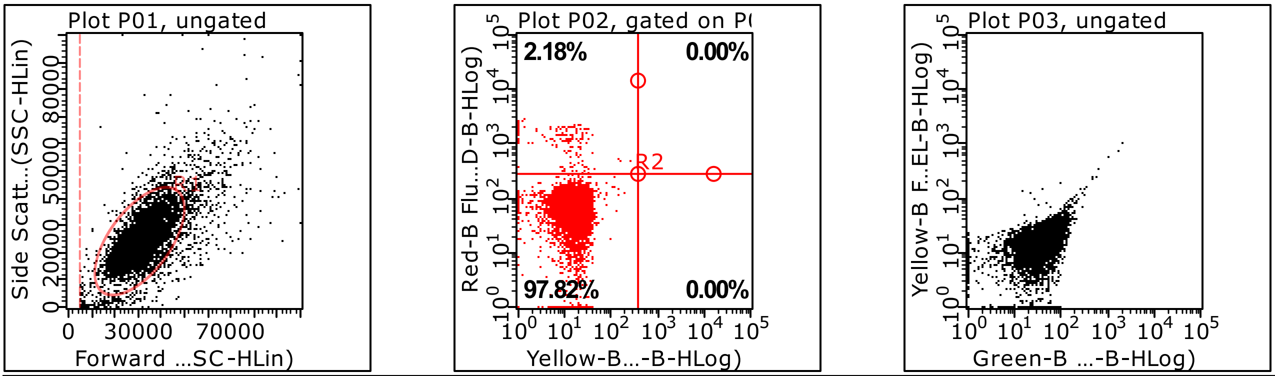

Well Number: A03 Sample ID: Myrislignan 70 ug/mL

File Name: D:/shf/2021-06-05_at_10-40-12pm-TOXOPLASMA-Myrislignan-apptosis.fcs

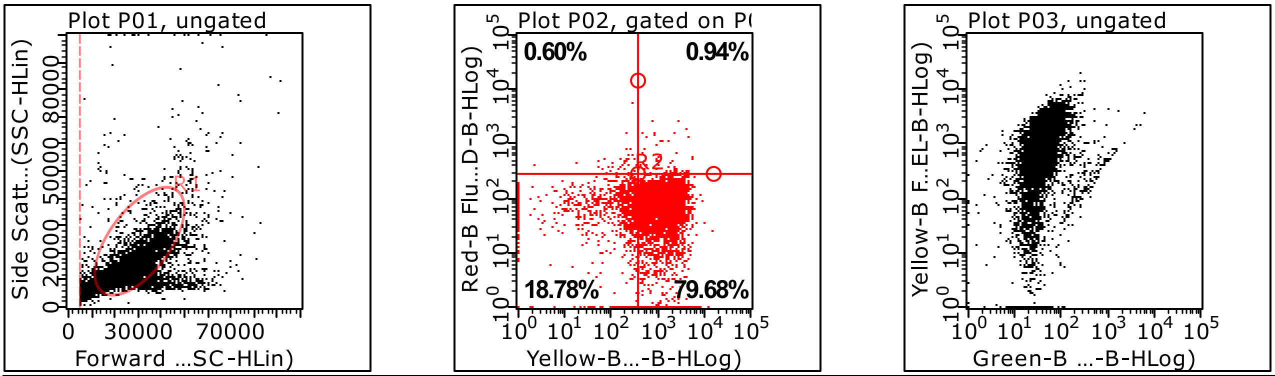

Well Number: A04 Sample ID: Myrislignan 50 ug/mL

File Name: D:/shf/2021-06-05_at_10-40-12pm-TOXOPLASMA-Myrislignan-apptosis.fcs

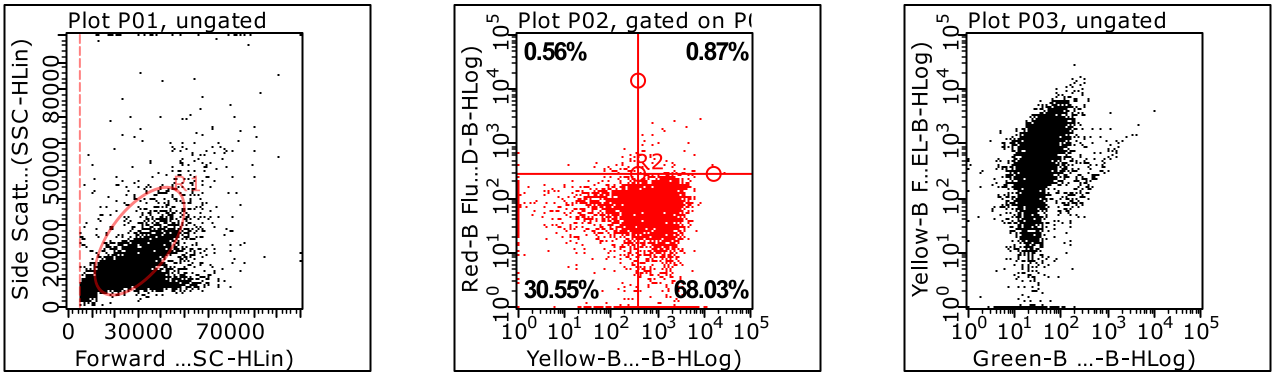

Well Number: A05 Sample ID: Myrislignan 32 ug/mL
File Name: D:/shf/2021-06-05_at_10-40-12pm-TOXOPLASMA-Myrislignan-apptosis.fcs

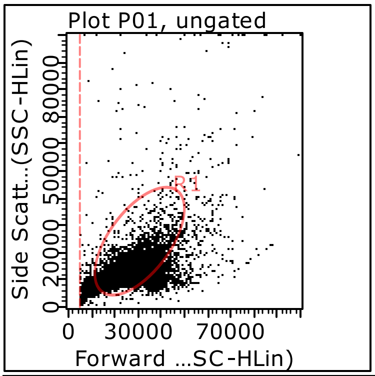

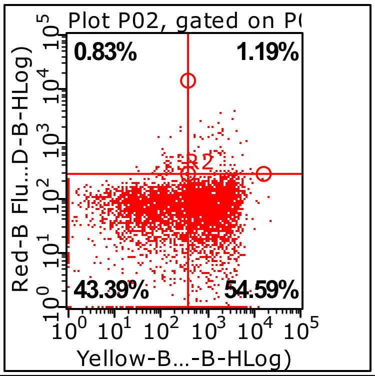

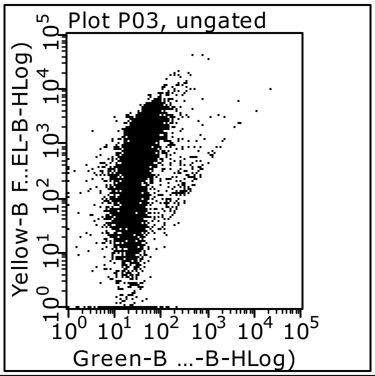

Well Number: A06 Sample ID: Myrislignan 0 ug/mL
File Name: D:/shf/2021-06-05_at_10-40-12pm-TOXOPLASMA-Myrislignan-apptosis.fcs

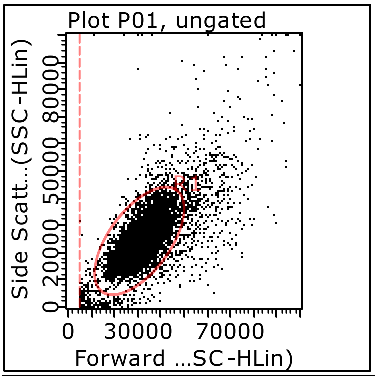

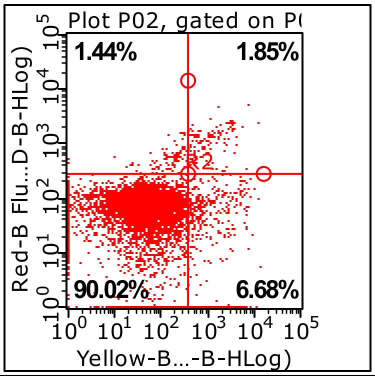

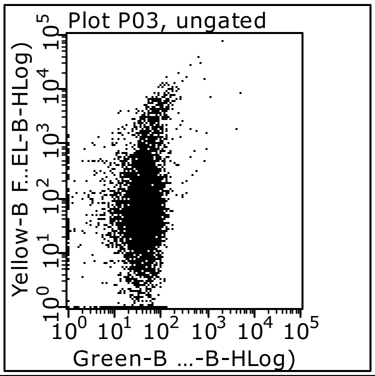

Well	Sample ID	Date	R2.Percent.UL Percent for R2 gated by P01.R1 (%)	R2.Percent.UR Percent for R2 gated by P01.R1 (%)	R2.Percent.LL Percent for R2 gated by P01.R1 (%)
A01	Annexin v	06.05.2021	0.04	0.03	92.27
A02	7AAD	06.05.2021	2.18	0.00	97.82
A03	Myrislignan 70 ug/mL	06.05.2021	0.60	0.94	18.78
A04	Myrislignan 50 ug/mL	06.05.2021	0.56	0.87	30.55
A05	Myrislignan 32 ug/mL	06.05.2021	0.83	1.19	43.39
A06	Myrislignan 0 ug/mL	06.05.2021	1.44	1.85	90.02

Well	R2.Percent.LR Percent for R2 gated by P01.R1 (%)
A01	7.66
A02	0.00
A03	79.68
A04	68.03
A05	54.59
A06	6.68
