# Supplementary material for: Myrislignan Induces Redox Imbalance and Activates Autophagy in Toxoplasma gondii
Source: Front Cell Infect Microbiol. 2021 Sep 3;11:730222. doi: 10.3389/fcimb.2021.730222 (PMC8447958; doi:10.3389/fcimb.2021.730222)
Supplement: Supplementary file 6 [file DataSheet_6.zip › Fig.6-raw data/2021-06-05_at_11-13-45pm-TOXOPLASMA--Myrislignan-2.pdf]

Well Number: A01 Sample ID: annexin v
File Name: D:/shf/2021-06-05_at_11-13-45pm-TOXOPLASMA--Myrislignan-apoptosis.fcs

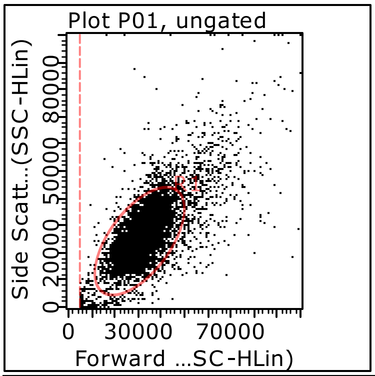

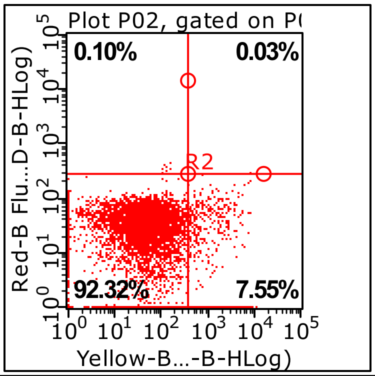

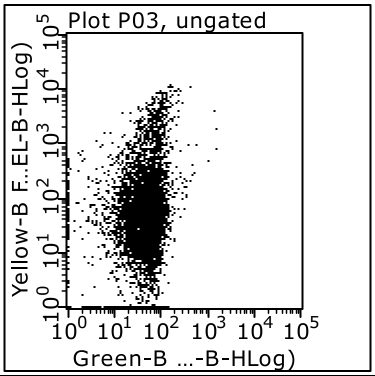

Well Number: A02 Sample ID: 7AAD
File Name: D:/shf/2021-06-05_at_11-13-45pm-TOXOPLASMA--Myrislignan-apoptosis.fcs

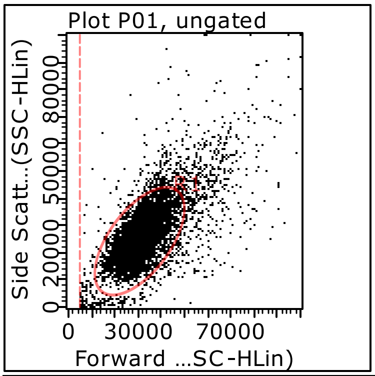

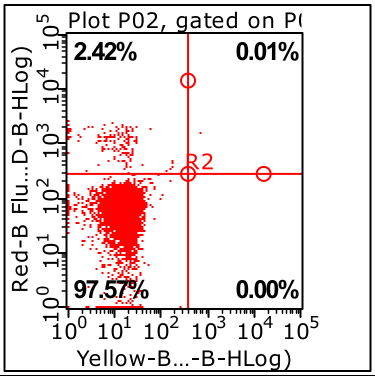

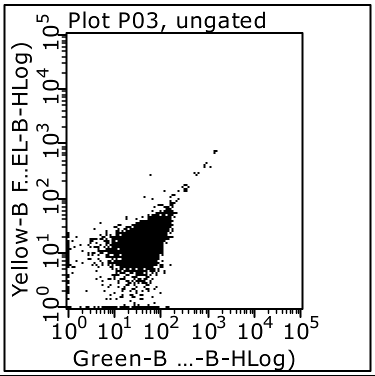

Well Number: A03 Sample ID: Myri-70ug/mL
File Name: D:/shf/2021-06-05_at_11-13-45pm-TOXOPLASMA--Myrislignan-apoptosis.fcs

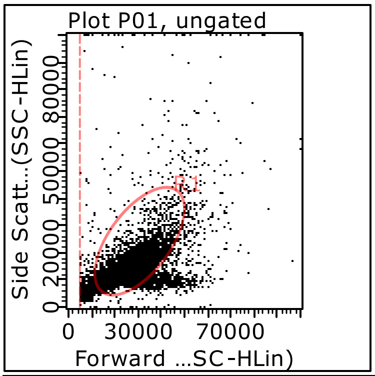

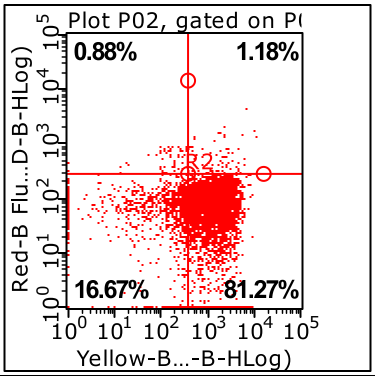

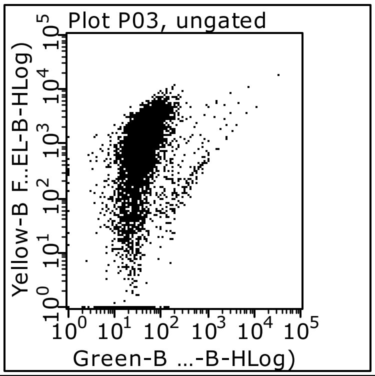

Well Number: A04 Sample ID: Myri-50ug/mL
File Name: D:/shf/2021-06-05_at_11-13-45pm-TOXOPLASMA--Myrislignan-apoptosis.fcs

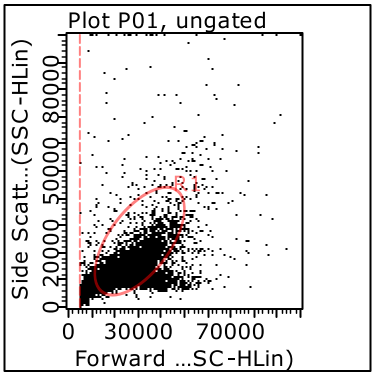

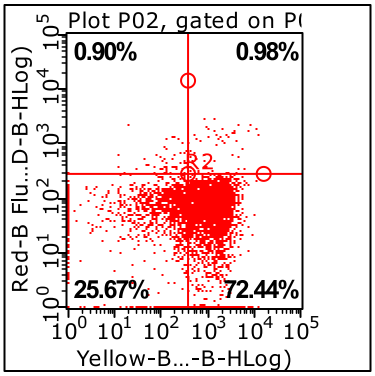

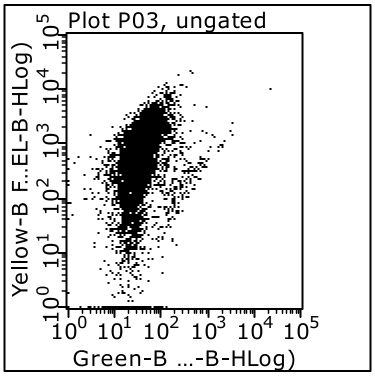

Well Number: A05 Sample ID: Myri-32ug/mL
File Name: D:/shf/2021-06-05_at_11-13-45pm-TOXOPLASMA--Myrislignan-apptosis.fcs

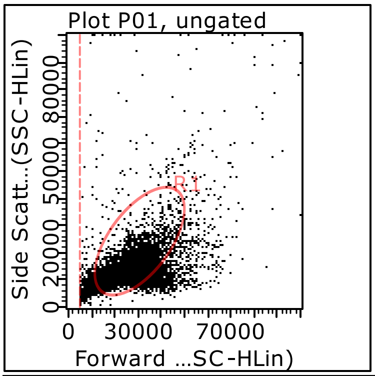

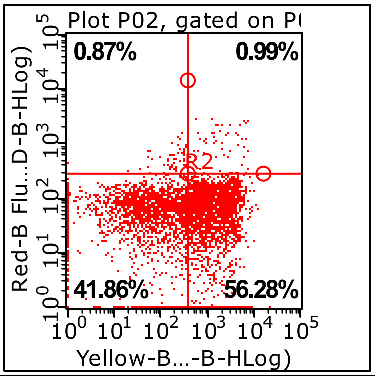

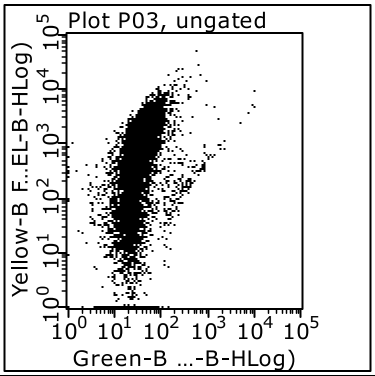

Well Number: A06 Sample ID: Myri-0ug/mL
File Name: D:/shf/2021-06-05_at_11-13-45pm-TOXOPLASMA--Myrislignan-apptosis.fcs

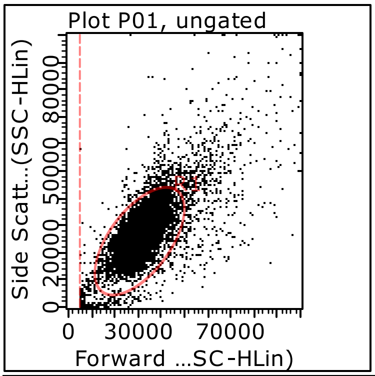

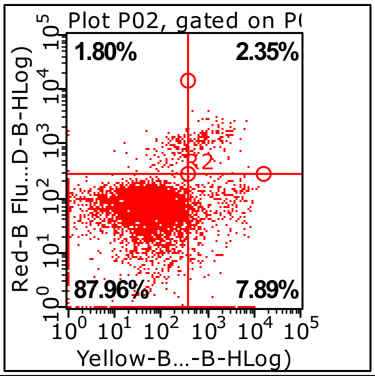

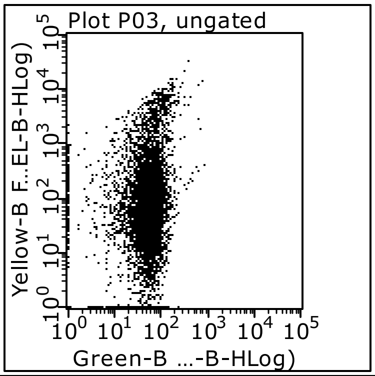

Well	Sample ID	Date	R2.Percent.UL Percent for R2 gated by P01.R1 (%)	R2.Percent.UR Percent for R2 gated by P01.R1 (%)	R2.Percent.LL Percent for R2 gated by P01.R1 (%)	R2.Percent.LR Percent for R2 gated by P01.R1 (%)
A01	annexin v	06.05.2021	0.10	0.03	92.32	7.55
A02	7AAD	06.05.2021	2.42	0.01	97.57	0.00
A03	Myri-70ug/mL	06.05.2021	0.88	1.18	16.67	81.27
A04	Myri-50ug/mL	06.05.2021	0.90	0.98	25.67	72.44
A05	Myri-32ug/mL	06.05.2021	0.87	0.99	41.86	56.28
A06	Myri-0ug/mL	06.05.2021	1.80	2.35	87.96	7.89
