# Supplementary material for: Myrislignan Induces Redox Imbalance and Activates Autophagy in Toxoplasma gondii
Source: Front Cell Infect Microbiol. 2021 Sep 3;11:730222. doi: 10.3389/fcimb.2021.730222 (PMC8447958; doi:10.3389/fcimb.2021.730222)
Supplement: Supplementary file 6 [file DataSheet_6.zip › Fig.6-raw data/2021-06-05_at_11-21-55pm-TOXOPLASMA-Myrislignan-3.pdf]

Well Number: A01
Sample ID: 7aad
File Name: D:/shf/2021-06-05_at_11-21-55pm-TOXOPLASMA-Myrislignan-apoptosis.fcs

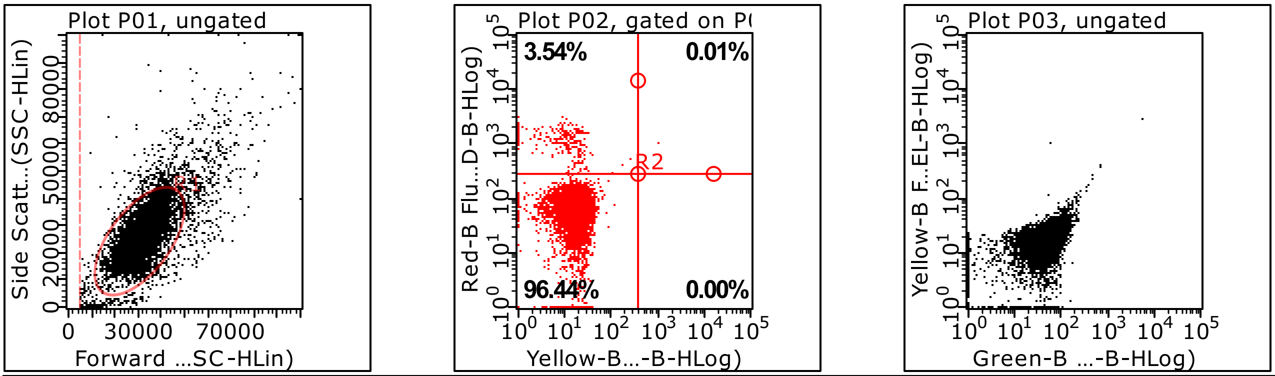

Well Number: A02
Sample ID: annexin v
File Name: D:/shf/2021-06-05_at_11-21-55pm-TOXOPLASMA-Myrislignan-apoptosis.fcs

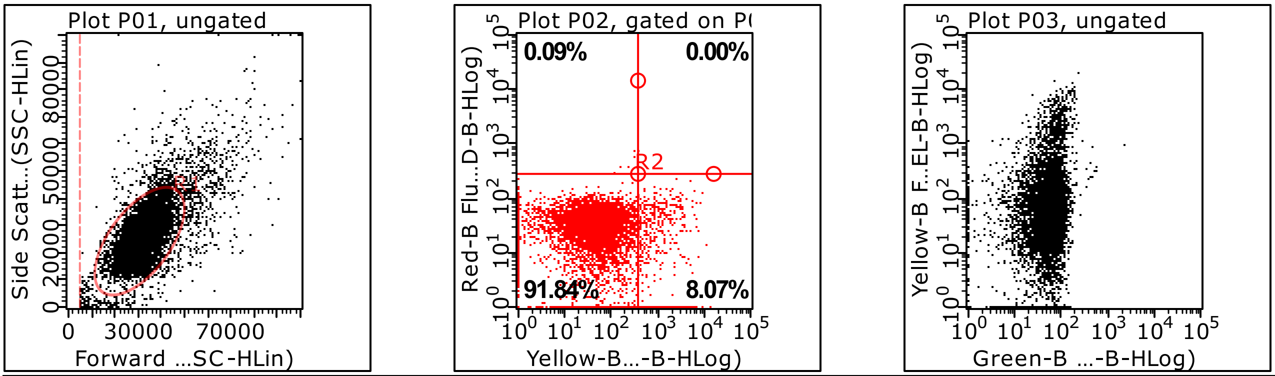

Well Number: A03
Sample ID: Myri 70 ug/mL
File Name: D:/shf/2021-06-05_at_11-21-55pm-TOXOPLASMA-Myrislignan-apoptosis.fcs

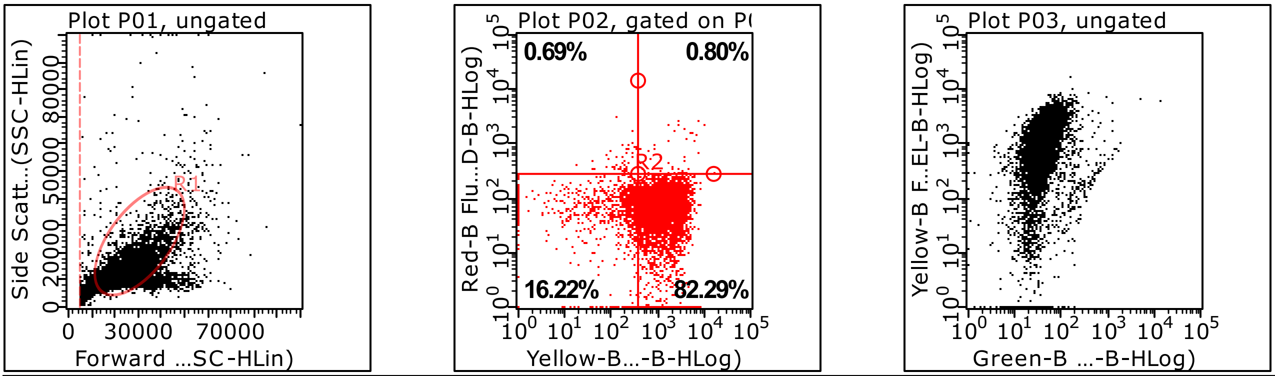

Well Number: A04
Sample ID: Myri 50 ug/mL
File Name: D:/shf/2021-06-05_at_11-21-55pm-TOXOPLASMA-Myrislignan-apoptosis.fcs

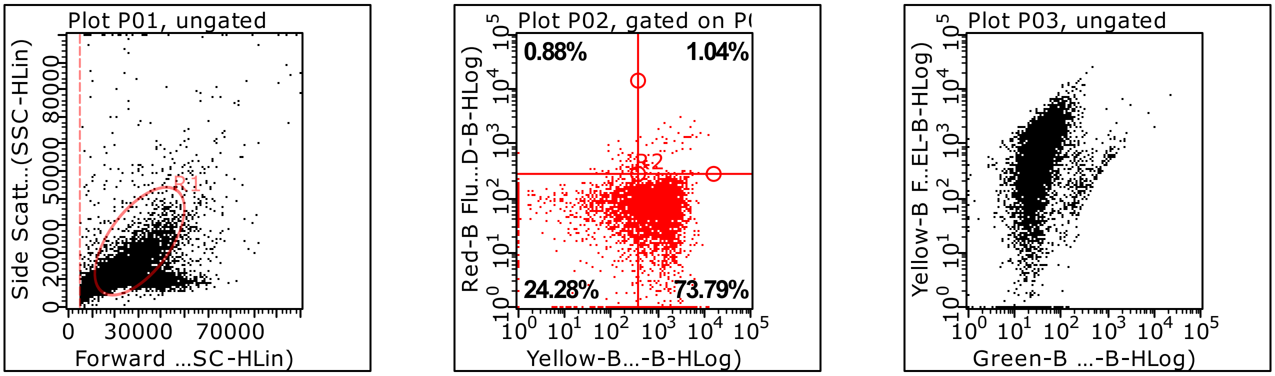

Well Number: A05 Sample ID: Myri 32 ug/mL
File Name: D:/shf/2021-06-05_at_11-21-55pm-TOXOPLASMA-Myrislignan-apptosis.fcs

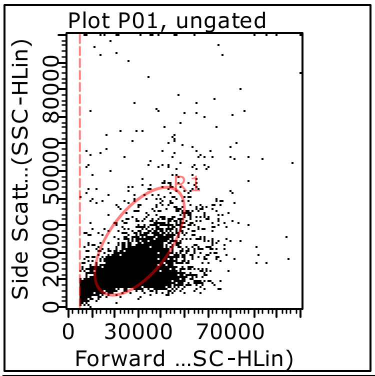

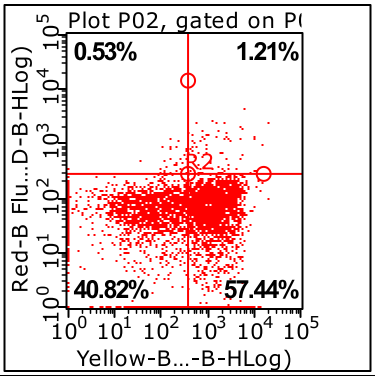

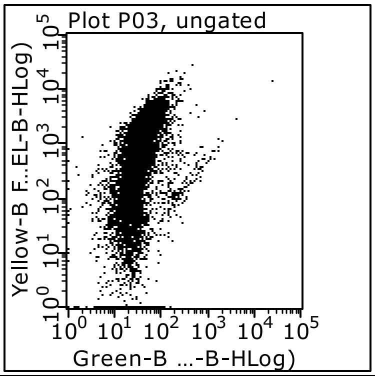

Well Number: A06 Sample ID: Myri 0 ug/mL
File Name: D:/shf/2021-06-05_at_11-21-55pm-TOXOPLASMA-Myrislignan-apptosis.fcs

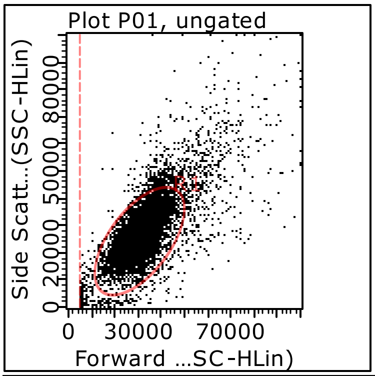

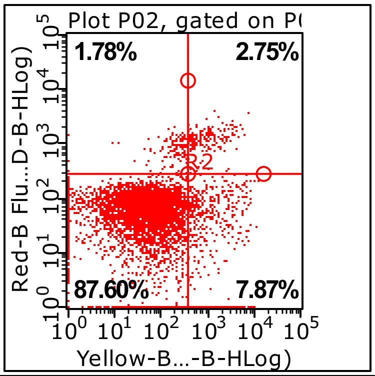

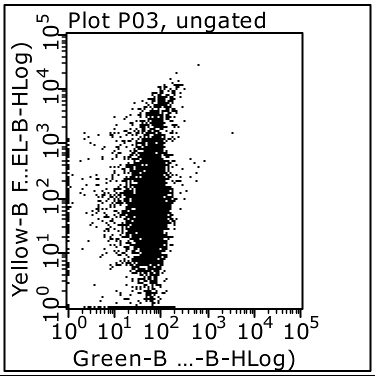

Well	Sample ID	Date	R2.Percent.UL Percent for R2 gated by P01.R1 (%)	R2.Percent.UR Percent for R2 gated by P01.R1 (%)	R2.Percent.LL Percent for R2 gated by P01.R1 (%)
A01	7aad	06.05.2021	3.54	0.01	96.44
A02	annexin v	06.05.2021	0.09	0.00	91.84
A03	Myri 70 ug/mL	06.05.2021	0.69	0.80	16.22
A04	Myri 50 ug/mL	06.05.2021	0.88	1.04	24.28
A05	Myri 32 ug/mL	06.05.2021	0.53	1.21	40.82
A06	Myri 0 ug/mL	06.05.2021	1.78	2.75	87.60

Well	R2.Percent.LR Percent for R2 gated by P01.R1 (%)
A01	0.00
A02	8.07
A03	82.29
A04	73.79
A05	57.44
A06	7.87
