# Supplementary material for: Myrislignan Induces Redox Imbalance and Activates Autophagy in Toxoplasma gondii
Source: Front Cell Infect Microbiol. 2021 Sep 3;11:730222. doi: 10.3389/fcimb.2021.730222 (PMC8447958; doi:10.3389/fcimb.2021.730222)
Supplement: Supplementary file 7 [file Table_1.docx]

**Supplementary Dataset S1**. The Q-PCR primers used for RNA-seq validation

| **Gene ID** | **Primers** |
| --- | --- |
| TGME49_203580 | 5'- AGTATGAAGGCGGATGGAATGAATGG -3'  5'- CGCTCGCTGCAAGATGAATAACAAC -3' |
| TGME49_330000 | 5'- GCGGCTTTTGGTTTGATGGAACTATC -3'  5'- TCAGGTACGATATGAAGTGGTGTTACG -3' |
| TGME49_278160 | 5'- CCTCAACTCATACGCGCTCC -3'  5'- GCTCAAACTGGTGAAGGGCA -3' |
| TGME49_281430 | 5'- CACGAATTTGCAGGCGTGTG -3'  5'- GGTGACGGCATTCTTGAGGG -3' |
| TGME49_249820 | 5'- TTCCACCTCCGAGATCCACC -3'  5'- AGATGGAGGGTCGAGACACG -3' |
| TGME49_231770 | 5'- TGATGGAGTGCATTCGTCGC -3'  5'- TCCTCGCGAACTCCACAGAT -3' |
