# Supplementary material for: Myrislignan Induces Redox Imbalance and Activates Autophagy in Toxoplasma gondii
Source: Front Cell Infect Microbiol. 2021 Sep 3;11:730222. doi: 10.3389/fcimb.2021.730222 (PMC8447958; doi:10.3389/fcimb.2021.730222)
Supplement: Supplementary file 8 [file Table_2.docx]

**Supplementary Dataset S2.** The specific binding proteins of *T. gondii* were captured by myrislignan as determined using SPR.

| **Entry** | **Protein names** | **Score** |
| --- | --- | --- |
| TGME49_309730 | thioredoxin reductase | 1810.95 |
| TGME49_281400 | phosphofructokinase domain-containing protein | 1798.63 |
| TGME49_265120 | rhoptry neck protein, putative | 1789.74 |
| TGME49_219630 | flavodoxin domain-containing protein | 1783.41 |
| TGME49_315730 | apical membrane antigen 1 protein | 1782.55 |
| TGME49_295590 | hypothetical protein | 1772.73 |
| TGME49_255970 | histone lysine methyltransferase SET/SUV39 | 1752.65 |
| TGME49_216680 | ankyrin repeat-containing protein | 1743.20 |
| TGME49_251620 | flap structure-specific endonuclease 1, putative | 1739.67 |
| TGME49_220340 | hypothetical protein | 1736.27 |
| TGME49_229690 | autophagy-related protein 7 atg7, putative | 1735.20 |
| TGME49_201890 | MORN repeat-containing protein | 1726.16 |
| TGME49_288050 | intraflagellar transport protein 172, putative | 1723.41 |
| TGME49_321530 | cathepsin CPL | 1715.73 |
| TGME49_311090 | ubiquitin carboxyl-terminal hydrolase | 1709.63 |
| TGME49_250880 | kinase, pfkB family protein | 1687.25 |
| TGME49_266760 | isocitrate dehydrogenase | 1667.03 |
| TGME49_310660 | Dullard family phosphatase domain-containing protein | 1646.72 |
| TGME49_233010 | cell-cycle-assocaited protein kinase ERK7, putative | 1645.14 |
| TGME49_315770 | cytochrome p450 superfamily protein | 1638.24 |
| TGME49_221330 | DNA gyrase/topoisomerase IV, A subunit domain-containing protein | 1631.93 |
| TGME49_265840 | hypothetical protein | 1622.90 |
| TGME49_201895 | hypothetical protein | 1596.30 |
| TGME49_221440 | RPGR, putative | 1592.70 |
| TGME49_316660 | cullin family protein | 1572.16 |
| TGME49_313140 | isocitrate dehydrogenase | 1232.53 |
| TGME49_209850 | RNA recognition motif-containing protein | 932.00 |
| TGME49_204440 | cyclic nucleotide-binding domain-containing protein | 914.34 |
| TGME49_213870 | UBA/TS-N domain-containing protein | 906.12 |
| TGME49_217760 | GTP-binding protein | 818.11 |
| TGME49_255260 | apical membrane antigen AMA1 | 799.44 |
| TGME49_263380 | Dullard family phosphatase domain-containing protein | 753.25 |
| TGME49_206590 | calcium-dependent protein kinase CDPK2A | 732.74 |
| TGME49_240890 | 6-phosphofructokinase | 715.00 |
| TGME49_314875 | RIC1 protein | 707.82 |
| TGME49_246920 | glutathione reductase | 701.33 |
| TGME49_289310 | cullin family protein | 694.49 |
| TGME49_310050 | RNA recognition motif-containing protein | 690.45 |
| TGME49_268000 | hypothetical protein | 683.24 |
| TGME49_214500 | ankyrin repeat-containing protein | 681.38 |
| TGME49_207820 | cell-cycle-associated protein kinase MAPK, putative | 649.11 |
| TGME49_261590 | ankyrin, putative | 638.72 |
| TGME49_257770 | histone lysine methyltransferase SET2 | 613.42 |
| TGME49_230490 | phosphatidylinositol-4-phosphate 5-kinase | 608.93 |
| TGME49_273200 | hypothetical protein | 607.49 |
| TGME49_213072 | Sec1 family protein | 601.01 |
| TGME49_239130 | Tyrosine kinase-like (TKL) protein | 592.44 |
| TGME49_249215 | hypothetical protein | 579.25 |
| TGME49_305030 | kinase, pfkB family protein | 564.97 |
| TGME49_208050 | ABC transporter, putative | 564.28 |
| TGME49_298990 | ferredoxin NADP+ oxidoreductase FNR | 543.11 |
| TGME49_233090 | XPG N-terminal domain-containing protein | 534.82 |
| TGME49_300100 | rhoptry neck protein RON2 | 518.82 |
| TGME49_312230 | DNA topoisomerase 2, putative | 501.84 |
| TGME49_312630 | anonymous antigen-1, putative | 492.49 |
| TGME49_256865 | WD repeat domain 35 family protein | 491.06 |
| TGME49_249670 | cathepsin B | 480.36 |
| TGME49_267360 | histone deacetylase SIR2-like | 433.29 |
